# SFRP2 modulates functional phenotype transition and energy metabolism of macrophages during diabetic wound healing

**DOI:** 10.3389/fimmu.2024.1432402

**Published:** 2024-10-11

**Authors:** Jiaqi Yang, Guorui Xiong, Huijuan He, Haili Huang

**Affiliations:** ^1^ Institute of Plastic Surgery, Affiliated Hospital of Guangdong Medical University, Zhanjiang, China; ^2^ Clinical Research Center, Affiliated Hospital of Guangdong Medical University, Zhanjiang, China

**Keywords:** diabetic wound healing, inflammation, macrophage functional phenotype transition, energy metabolism, SFRP2

## Abstract

Diabetic foot ulcer (DFU) is a serious complication of diabetes mellitus, which causes great health damage and economic burden to patients. The pathogenesis of DFU is not fully understood. We screened wound healing-related genes using bioinformatics analysis, and full-thickness skin injury mice model and cellular assays were used to explore the role of target genes in diabetic wound healing. SFRP2 was identified as a wound healing-related gene, and the expression of SFRP2 is associated with immune cell infiltration in DFU. *In vivo* study showed that suppression of SFRP2 delayed the wound healing process of diabetic mice, impeded angiogenesis and matrix remodeling, but did not affect wound healing process of control mice. In addition, suppression of SFRP2 increased macrophage infiltration and impeded the transition of macrophages functional phenotypes during diabetic wound healing, and affected the transcriptome signatures-related to inflammatory response and energy metabolism at the early stage of wound healing. Extracellular flux analysis (EFA) showed that suppression of SFRP2 decreased mitochondrial energy metabolism and increased glycolysis in injury-related macrophages, but impeded both glycolysis and mitochondrial energy metabolism in inflammatory macrophages. In addition, suppression of SFRP2 inhibited wnt signaling-related genes in macrophages. Treatment of AAV-SFRP2 augmented wound healing in diabetic mice and demonstrated the therapeutic potential of SFRP2. In conclusions, SFRP2 may function as a wound healing-related gene in DFU by modulating functional phenotype transition of macrophages and the balance between mitochondrial energy metabolism and glycolysis.

## 1 Introduction

Diabetic foot ulcer (DFU) is a serious complication of diabetes mellitus. The healing and prognosis of diabetic foot ulcers are the focus of clinical attention (1). DFU is characterized by persistent chronic inflammation, impaired angiogenesis, and matrix remodeling (2). Most of the traditional treatment methods cannot achieve satisfactory healing. Recent study reveals that a therapy targeting macrophages shows better healing efficacy in DFU (3).

Abnormalities of inflammatory response contribute critically to the diabetic chronic refractory wounds, which requires the participation of immune cell populations such as macrophages (4). Macrophages involved in the diabetic wound healing include skin-tissue-resident macrophages and monocyte-derived macrophages, and the latter is considered to play a dominant role in wound healing (4, 5). Macrophages may be polarized to inflammatory phenotype at the early stage, in which inflammatory macrophages eliminate pathogen and cell debris. At the later stage of wound healing, inflammatory macrophages convert to pro-healing anti-inflammatory macrophages (5). The delayed transition of inflammatory phenotype to anti-inflammatory phenotype has been implicated in persistent inflammation of diabetic wounds (2). Recent studies revealed distinct metabolic signature of these two functional phenotype. The metabolic immunomodulation of macrophage functional phenotype is critical to inflammatory response (2). The inflammatory macrophages are characterized by increased aerobic glycolysis and an uncoupling of the TCA cycle at both citrate and succinate (6). The oxidative phosphorylation and intact TCA cycle are observed in anti-inflammatory phenotype (6).

Secreted Frizzled-related protein 2 (SFRP2) is a member of the SFRP family that contains a cysteine-rich domain homologous to the putative wnt-binding site of Frizzled proteins (7). SFRPs act as soluble modulators of wnt signaling through direct interaction with wnts (7). SFRP2 has been implicated in multiple physiological and pathological processes, including carcinogenesis (8–14), myogenesis and cardiomyopathy (15–18), retinal development (19), dental tissue regeneration (20–24), and airway inflammation (25). In the field of skin research, SFRP2 plays a role in adaptation of epidermis to mechanical stretching (26), hypertrophic scars (27), and systemic sclerosis (28). In the current study, we identified SFRP2 as a wound-healing-related gene in DFU. *In vitro* and *in vivo* studies were applied to explore the role of SFRP2 in diabetic wound healing.

## 2 Materials and methods

### 2.1 Data acquisition and processing

Four datasets were obtained from the Gene Expression Omnibus (GEO) database. GSE80178 and GSE134431 were used as testing datasets; GSE143735 and GSE97615 were used as validation dataset. For data processing, the R software “biomaRt” package was used to convert probe ID and gene name. If there were multiple probe IDs for one gene, the average value was retained. The probe ID data without corresponding gene name and the probe ID data with missing values were removed. Boxplot and cluster analysis were used to analyze the homogeneity of data. Given that the two datasets came from different experimental groups and belonged to different platforms and different types of data, for the sake of caution, we merge the data after separate analysis. The differential expressed genes (DEGs) obtained from two separate datasets were intersected. The R software “limma” package was used to analyze the differences between the two datasets, and the threshold of DEGs was set according to the adjusted p-value [P.adj< 0.05 and |log2FC|>2(FC:fold change)]. The “venn” package was used to intersect DEGs between two separate datasets.

### 2.2 Functional enrichment analysis

The R software “Bioiconductor” and “GOplot” packages were used to analyze the intersection DEG and perform GO enrichment, which included sub-function (MFs), biological process (BP), and cellular component (CC) and KEGG pathway enrichment analysis.

### 2.3 Protein interaction network construction and key gene identification (PPI)

To further identify the direct or indirect interacting proteins associated with DEGs, a PPI network was constructed using STRING version 11.5. The confidence score was set to >0.700 for network construction. Network visualization was performed by using Cytoscape 3.9.2. Cyto-Hubba plug-in was used to identify hub genes.

### 2.4 Immune cell infiltration analysis

The immune cell infiltration of GSE134431 was analyzed by R software “bseqsc” and “CIBERSORT” packages. The “ggplot2,” “pheatmap,” and “Complex Heatmap” packages were used to generate the heat map and bar map of immune cell infiltration The “ggcorrplot,” “corrplot,” “RColorBrewer,” and “grDevices” packages were used to visualize the correlation between immune cell infiltration and SFRP2.

### 2.5 Assessment of diabetic wound healing

The db/db C57BL/Ks mice and wild-type (WT) BLKS-leprdb mice were obtained from GemPharmtech Co., Ltd (Nanjing, China). The wild-type (WT) BLKS-leprdb mice were used as control mice for db/db C57BL/Ks mice. Animal protocols followed the Guide for the Care and Use of Laboratory Animals issued by the US National Institutes of Health (NIH). Extensive efforts were made to minimize both animal use and animal suffering. Dorsal full-thickness skin wounds were created by 6-mm punch biopsies and were randomly treated with control siRNAs (NC) and siRNAs targeting SFRP2. The 2′OMe-modified siRNAs were obtained from Guangzhou RiboBio Co., Ltd (Guangzhou, China) Three injection sites were set around each wound, and 2 nM siRNAs (for each injection site) was subcutaneously injected to wound tissues. For the evaluation of the effects of AAV-SFRP2 on wound healing, the db/db mice were treated with AAV-SFRP2 or AAV control. Dorsal full-thickness skin wounds were created after 14 days. The conditions of the wounds were observed and recorded. Each entire wound was photographed by digital camera at 3-day intervals after anesthesia when placed against the backdrop of a scaled ruler. Photographs were then analyzed with ImageJ software. The percentage of wound closure that represents the percentage of wound reduction from the original wound size was calculated according to the following formula: wound closure = (1 − SA)/S0 × 100%. (S0 represents the origin area on day 0, and SA represents the wound area on day A, accordingly).

### 2.6 Histological and immunofluorescence analysis

The whole wound bed of each mice was obtained for histological and immunofluorescence analysis. The tissues were fixed with 4% paraformaldehyde and stored at 4°C. After a series of alcohol dehydration and paraffin embedding, the samples were sectioned into 4-μm-thick sections perpendicularly before further staining. The status of re-epithelialization was analysis by hematoxylin and eosin staining (HE), and the degree of collagen maturity was evaluated by Masson’s trichrome staining. The calculation of un-healed wound area, scar length and thickness, density of collagen fiber, and positive ratio of indicated proteins were done by ImageJ software.

For immunohistochemical (IHC) and immunofluorescence (IF) staining, the paraffin-embedded tissue sections were dewaxed and rehydrated. Hydrogen peroxide solution (3%) was used to neutralize endogenous peroxidase activity. Normal goat serum (10%) was used to block nonspecific binding. The sections were incubated with indicated primary antibodies overnight at 4°C and secondary antibody at room temperature for 1 h. For IHC staining, the DAB Horseradish Peroxidase Color Development Kit (Beyotime Biotechnology, Shanghai, China) was used to visualize protein in wound tissues. For IF staining, proteins were visualized using a FV3000 Olympus laser scanning confocal microscope with a ×20 objective. The relative fluorescence intensity was calculated by the fluorescent module of ImageJ software. Antibodies used in this study are listed in **Supplementary Table S1**.

### 2.7 RNA-sequence analysis

RNA-sequence analysis was performed to analyze the changes of transcriptome in wound tissues of mice and macrophages. Total RNA was extracted from wound tissues and macrophages using TRIzol^®^ Reagent according to the manufacturer’s instructions (Magen). RNA samples were detected based on the A260/A280 absorbance ratio with a Nanodrop ND-2000 system (Thermo Scientific, Waltham, MA, USA), and the RIN of RNA was determined by an Agilent Bioanalyzer 4150 system (Agilent Technologies, CA, USA). Sequencing was performed with an Illumina Novaseq 6000/MGISEQ-T7 instrument. Differential expression analysis was performed using the DESeq2 (http://bioconductor.org/packages/release/bioc/html/DESeq2.html); DEGs with |log2FC|>1 and adjusted p <0.05 were considered to be significantly differently expressed genes. Gene Ontology (GO) terms and Kyoto Encyclopedia of Genes and Genomes (KEGG) pathways with adjusted p < 0.05 were considered significantly enriched.

### 2.8 Wound cell isolation and cell sorting

Wounds were harvested, chopped, and digested with Liberase TL (Sigma-Aldrich; Cat. No. 5401020001)/DNase I (Sigma-Aldrich; Cat. No. 9003-98-9) and plunged through a 100-μm nylon filter to yield a single-cell suspension. Cells were stained with a Fixable LIVE/DEAD viability dye (Molecular Probes by Life Technologies; Ref No. L34959; 1:1,000 dilution) to exclude dead cells. Monoclonal antibodies for surface staining included: APC-cyanine7-conjugated anti-CD11b (diluted 1:300, Thermo Fisher Scientific, A15390), and PE–Cyanine5-conjugated anti-F4/80 (diluted 1:500, Thermo Fisher Scientific, 15-4801-82). Cells were analyzed using a FACSCanto II flow cytometer (BD) and sorted using a FACSAria III cell sorting system (BD Biosciences) equipped with FACSDiva Version 6.1.1 software (BD Biosciences). FACS data were further analyzed by FlowJo Version 10.7.1 (FlowJo).

### 2.9 Extracellular flux analysis

Extracellular flux analysis (EFA) analysis was performed to examine the energy metabolism of macrophages as previously described (6). Cells were seeded in an Agilent seahorse XF24 cell culture microplate and cultured with the prepared XF medium. The cell culture microplate was placed in a 37°C non-CO^2^ incubator for 1 h prior to the assay. Load oligomycin into port A, FCCP to port B, and rotenone/antimycin to port C of the hydrated sensor cartridge. The oxygen consumption rate (OCR) and extracellular acidification rate (ECAR) of macrophages were then determined after sequentially administration of the following reagents (final concentration): 1.0 mM oligomycin (Selleck, S1478), 0.75 mM FCCP (Sigma-Aldrich, C2920), and 100nM rotenone (Sigma-Aldrich, R8875) + 1.0 mM antimycin (Biovision, 2247-50) using the extracellular flux analyzer.

### 2.10 Assessment of mitochondrial mass and reactive oxygen species

CD11b^+^F4/80^+^ wound macrophages were flow cytometry-sorted and incubated with MitoTracker™ Green. Mean fluorescence intensity in the FITC channel in the gate of CD11b^+^F4/80^+^ wound cells was used as a measure of mitochondrial mass. For the measurement of reactive oxygen species (ROS), cells were incubated with a DCFH probe; mean fluorescence intensity in the FITC channel in the gate of CD11b^+^F4/80^+^ wound cells was used as a measure of ROS. At least two independent experiments were performed.

### 2.11 Cell culture and siRNA transfection in RAW264.7 cells

The RAW264.7 cells were obtained from the American Type Culture Collection (ATCC). The RPMI 1640 medium (Gibco, C11875500BT) was used and supplemented with of 10% fetal bovine serum. Cells were cultured at 37°C with 5% CO_2_. The siRNA-targeted SFRP2 were obtained from Guangzhou RiboBio Co., Ltd. Cell transfection was performed using Lipofectamine 3000 (Invitrogen) according to the product operation manual. The transfection system for the six-well plate contained 10 nM siRNA and 5 μl Lipofectamine 3000. Cells (3×10^5^) were seeded into the well 1 day before transfection. siRNAs were mixed with Lipofectamine 3000 according to the manufacturer’s protocol. The mixture was incubated with cells for 6 h. The culture media were discarded and replaced with fresh media. Cells were maintained for 48 h at 37°C and then subjected into further examinations. For induction of inflammatory phenotype, RAW264.7 cells were treated with LPS (100 ng/ml) and IFN-γ (20 ng/ml) for 24 h.

### 2.12 Statistical analysis

GraphPad Prism software version 6.0 was used to analyze the data. Data are presented as the mean ± SD. Differences between two groups were analyzed by Student’s t-test. All data are representative of at least three independent experiments. A p-value of ≤0.05 was considered significant.

## 3 Results

### 3.1 Deficiency of SFRP2 may be associated with impaired healing power in DFU

**Figure 1:**
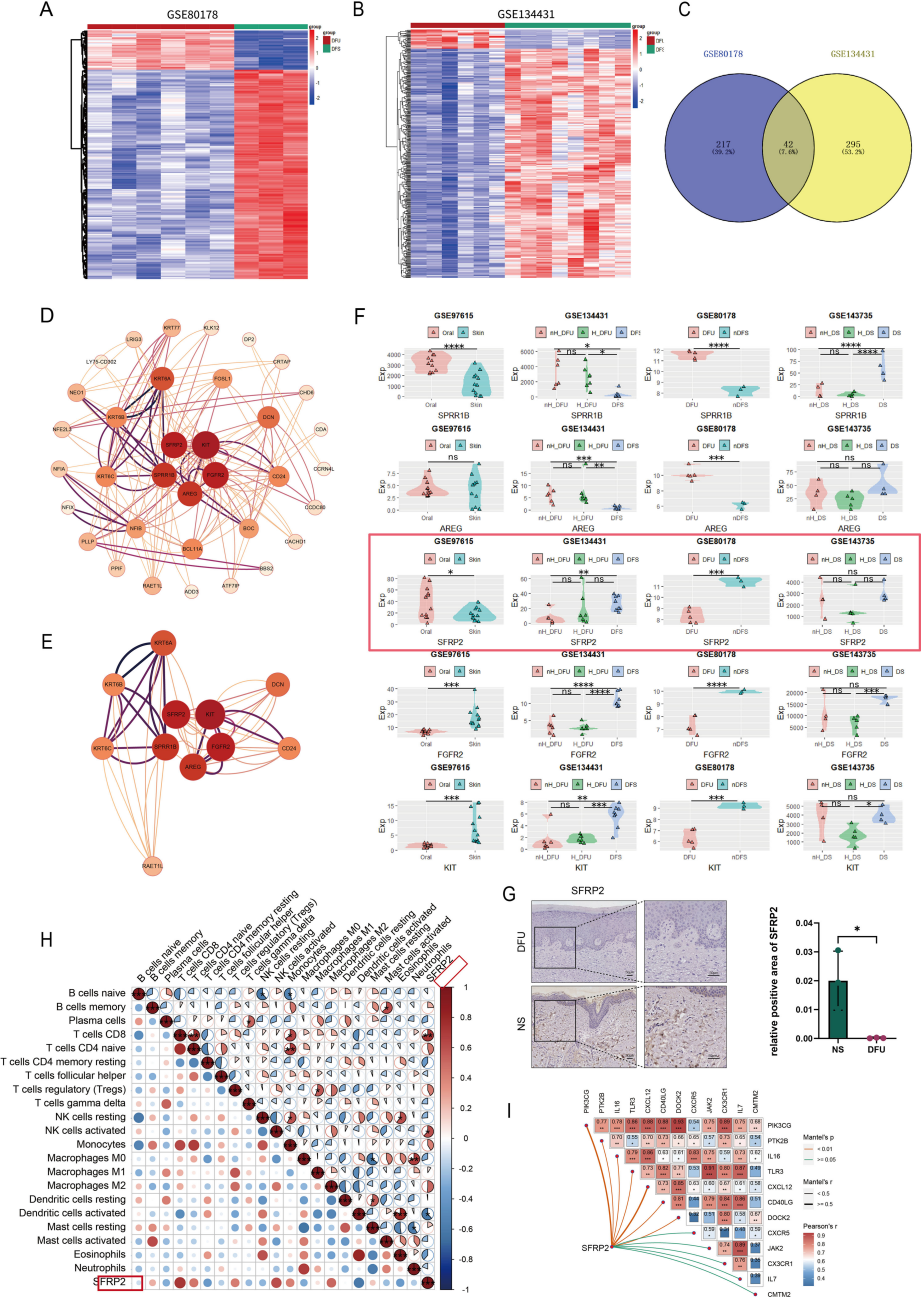
SFRP2 is a wound-healing-related gene in DFU. **(A, B)** Heat map of datasets GSE80178 and GSE134431. **(C)** Venn diagram of DEGs obtained from GSE80178 and GSE13443 (Adj.P value<0.05, Log2FoldChange<−2/Log2FoldChange>2). **(D)** PPI network of DEGs. **(E)** Central genes of PPI network. **(F)** The expression levels of central genes in GEO datasets with different tissue sources. GSE97615: Oral, oral mucosa; Skin, skin. GSE134431: H-DFU, healer-diabetic foot ulcer; nH-DFU, non-healer-diabetic foot ulcer. Skin, diabetic foot skin. GSE80178: DFU, diabetic foot ulcer; n-DFS, non-diabetic foot skin. GSE134735: DS, diabetic forearm skin (no ulcer); H_DS, healer-diabetic forearm skin (ulcer). nH_DS, non-healer-diabetic forearm skin (ulcer). **(G)** IHC staining of SFRP2 in DFU and normal skin tissues (NS). **(H)** Correlation between SFRP2 expression levels and immune cells infiltration of datasets GSE134431. **(I)** Correlation between SFRP2 expression levels and inflammation-related genes. *, P<0.05; **, P<0.01; ***, P<0.001; ****, P<0.0001. ns, non-significant.

Two datasets, GSE80178 and GSE134431, were used to screen DEGs related to diabetic wound healing (**Figures 1A, B**). These data were homogenized and analyzed by clustering (**Supplementary Figure S1**). A total of 42 overlapped DEGs were obtained (**Figure 1C**). PPI analysis was constructed to show the central DEGs of diabetic wound healing. SFRP2, KIT, FGFR2, AREG, and SPRR1B were considered as the most crucial DEGs in the PPI network (**Figures 1D, E**). FGFR2 and AREG have been implicated in wound healing and tissue regeneration (29, 30), which demonstrates the effectiveness of our screening method. The expression of SFRP2, KIT, FGFR2, AREG, and SPRR1B was verified in four datasets, GSE97615, GSE134431, GSE80178, and GSE143735. The sample types of these datasets were divided as oral mucus, skin tissues, non-healing diabetic foot ulcers (nH-DFU), healing diabetic foot ulcers(H-DFU), diabetic foot ulcer (DFU), non-diabetic foot skin (nDFS), non-healing diabetic forearm skin ulcer (nH-DS), healing diabetic forearm skin ulcer (H-DS), and diabetic forearm skin (DS). We found that SFRP2 was upregulated in the oral mucus compared with skin tissues (GSE97615) and downregulated in nH-DFU compared with DFS (GSE134431) (**Figure 1F**). No significant difference was observed between H-DFU and DFS, or nH-DFU (GSE134431). In addition, SFRP2 was downregulated in DFU compared with nDFS (GSE80178). No significant difference was observed between nH-DS and H-DS or DS (**Figure 1F**). The downregulation of SFRP2 in nH-DFU and upregulation in the oral mucus, which shows stronger healing power, suggested that expression of SFRP2 associates with healing power and the deficiency of SFRP2 may be associated with impaired healing power in nH-DFU (**Figure 1F**). Based on these findings, we considered SFRP2 as the most potential wound-healing-related genes. IHC staining confirmed that SFRP2 was downregulated in DFU compared with normal skin tissues (**Figure 1G**). We also examined the expression profile of SFRP2 in the main cell populations (endothelial cells, fibroblasts, neutrophils, and macrophages) of skin tissues using IF staining. The endothelial cells, fibroblasts, neutrophils, and macrophages were marked by their corresponding markers CD31, α-SMA, ly6G, and CD11b. As shown in **Supplementary Figure S2**, the accumulation of SFRP2 proteins in db/db mice were lower than those of WT mice. The SFRP2 proteins mainly distributed in endothelial cells and fibroblasts. A small fraction of SFRP2 proteins was located in macrophages and neutrophils.

We also explored the correlation between the expression of SFRP2 and immune cells infiltration using dataset GSE134431. As shown in **Figure 1H**, the expression of SFRP2 correlate with CD8^+^ T cells infiltration, activated NK cells infiltration, and activated dendritic cells infiltration in DFU, suggesting that SFRP2 may play a role in immune cells infiltration during wound healing. Moreover, the expression of SFRP2 correlate with several inflammation-related genes such as OIK3CG, PTK2B, IL-16, TLR3, CXCL12, CD40LG, DOCK2, JAK2, CXC3R1, IL-7, and CMTM2 (**Figure 1I**). This finding indicates SFRP2 may play a role in the regulation of inflammation during wound healing.

### 3.2 Suppression of SFRP2 impedes wound healing of diabetic mice

**Figure 2:**
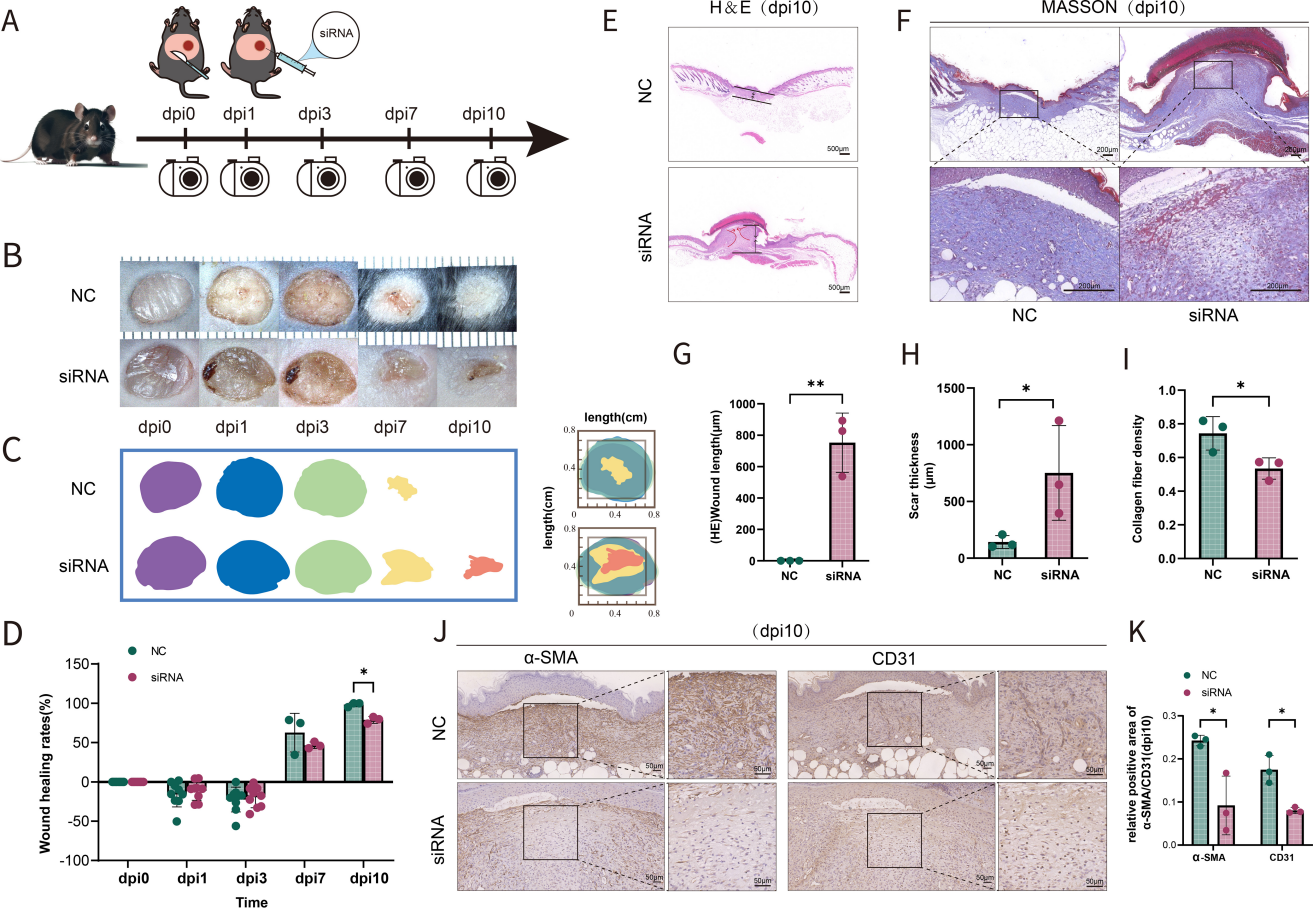
Suppression of SFRP2 impedes wound healing of diabetic mice. **(A)** Schematic diagram of the intervention of SFRP2 siRNAs in diabetic wound healing. The db/db BLKS mice were treated with SFRP2 siRNA or control siRNA (defined as NC), and wound healing were evaluated at indicated time points. **(B)** Representative images of wound healing. **(C, D)** Wound healing rate of db/db mice. **(E)** HE staining of wound tissue, bar=500 μm. **(F)** MASSON staining of wound tissues, bar = 200 μm. **(G, H)** The statistics of unhealed wound length and thickness. **(I)** The statistics of collagen deposition based on MASSON staining. **(J)** IHC staining of CD31 and α-SMA. Bar = 50 μm. **(K)** The statistics of angiogenesis and matrix deposition based on IHC staining. Data presented as means ± SD. *p<0.05; **p<0.01.

**Figure 3:**
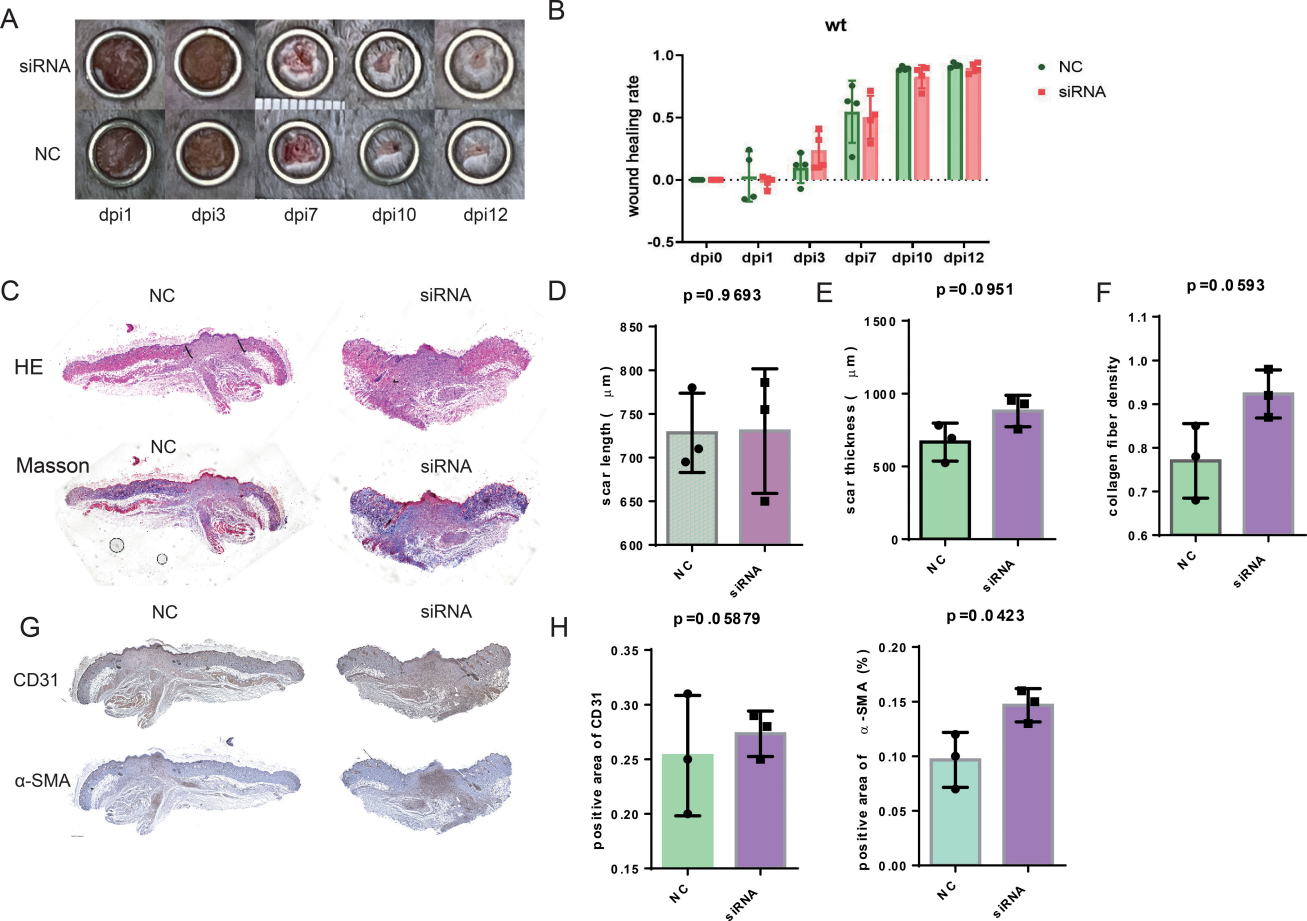
Suppression of SFRP2 does not change healing process in control mice. The wild type (WT) BLKS-leprdb mice were treated with SFRP2 siRNA or NC. The wound healing was evaluated at indicated time points. **(A)** Representative images of wound healing. **(B)** The statistics of wound healing rate. **(C)** Representative images of HE and MASSON staining. Bar = 500 μm. **(D, E)** The statistics of scar length and thickness based on HE staining. **(E)** The statistics of collagen fiber density based on MASSON staining. **(G)** IHC staining of CD31 and α-SMA. Bar = 500 μm. **(H)** The statistics of angiogenesis and matrix deposition based on IHC staining. Data presented as means ± SD.

To investigate the possibility that the deficiency of SFRP2 contributed to impaired diabetic wound healing, a model of full-thickness excisional skin injury was used. The db/db BLKS mice were treated with SFRP2 siRNAs or control siRNAs (defined as NC), and wound healing was evaluated (**Figure 2A**). Decreased accumulation of SFRP2 was observed in wound tissues of mice treated with siRNAs (**Supplementary Figure S3**). The wild-type (WT) BLKS-leprdb mice were used as control mice. We found that SFRP2 siRNAs delayed the wound healing process (**Figures 2B–D**), impeded re-epithelialization, and caused hyperplastic scar, characterized by HE staining (**Figures 2E, G, H**). In addition, SFRP2 siRNAs reduced collagen deposition characterized by MASSON staining (**Figures 2F, I**), and impeded angiogenesis and matrix remodeling, as demonstrated by decreased CD31 positive vessel-like structures and α-SMA positive extracellular matrix area (**Figures 2J, K**). SFRP2 siRNAs did not change the wound healing rate in WT mice (**Figures 3A, B**). Hyperplastic scar and increased collagen deposition were observed in WT mice treated with siRNAs, but the statistics analysis showed no difference (**Figures 3C–F**). SFRP2 siRNAs increased α-SMA positive extracellular matrix area, but did not change the status of angiogenesis in wound tissues of WT mice (**Figures 3G, H**). Collectively, these data indicate that the suppression of SFRP2 impeded diabetic wound healing.

### 3.3 Suppression of SFRP2 increases macrophage infiltration and impedes the functional phenotypes transition

**Figure 4:**
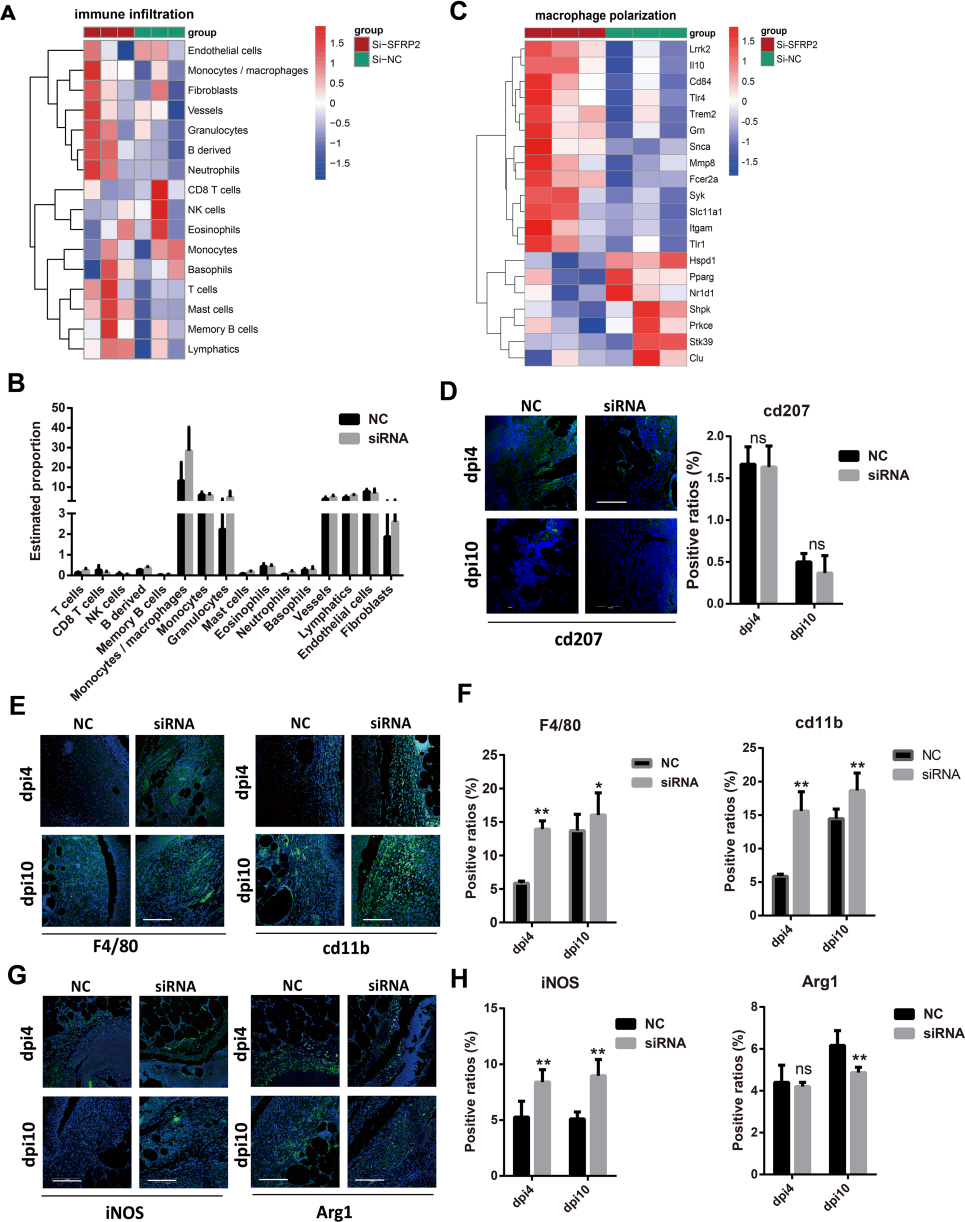
Suppression of SFRP2 increases macrophage infiltration and impedes functional phenotypes transition. Wound tissues were collected at dpi4 and subjected to RNA-seq analysis to explore the changes of transcriptome. The status of immune cell infiltration was analyzed using CIBERSORT algorithm. **(A)** Heat map of immune cell infiltration based on RNA-seq analysis of wound tissues. **(B)** The ratios of immune cells. **(C)** Heat map of macrophage polarization-related genes. **(D)** IF staining of CD207. **(E)** IF staining of F4/80 and CD11b, bar = 200 μm. **(F)** The statistics of F4/80 and CD11b-positive cells. **(G)** IF staining of iNOS and Arg1, bar = 200 μm. **(H)** The statistics of iNOS and Arg1-positive cells. Data presented as means ± SD. *p<0.05; **p<0.01. ns, non-significant.

We further investigate the effects of SFRP2 siRNAs on the immune cells infiltration of wound tissues. RNA-seq analysis was used to explore the changes of transcriptome in wound tissues at the early inflammatory phase [4 days post-injury (dpi4)]. As shown in **Figures 4A, B**, monocytes/macrophages were the major immune cells in wound tissues, and SFRP2 siRNAs increased macrophage infiltration. Neutrophils and macrophages are the two main inflammatory cell populations in the inflammatory stage of wound healing (31). Neutrophils are the first inflammatory cell population that arrived at the wound site and initiate the inflammatory response (31). Macrophages arrive second following neutrophils and eliminate neutrophils (5). Macrophages of the wound site consist of two sub-populations, tissue-resident macrophages and bone marrow-derived macrophages (32). We found that SFRP2 siRNAs neither affect the ratio of neutrophils (marked by ly6G) at dpi4 (**Supplementary Figure S4**) nor the ratio of tissue-resident macrophages as marked by CD207 throughout the whole healing process (dpi4 and dpi10) (**Figure 4D**). However, SFRP2 siRNAs increased the ratio of bone marrow-derived macrophages, marked by F4/80 and CD11b, both at dpi4 and dpi10 (**Figures 4E, F**).

Macrophages polarization contributes critically to inflammatory response (5). We further explored the effect of SFRP2 siRNAs on macrophage-polarization-related genes based on the RNA-seq analysis at dpi4. As shown in **Figure 4C** and **Supplementary Figure S6D**, suppression of SFRP2 affects most of macrophage-polarization-related genes and inflammation-related genes. IF staining was applied to define the inflammatory and anti-inflammatory phenotype macrophages in wound tissues using iNOS and Arg1 staining, respectively. Data showed that SFRP2 siRNAs increased inflammatory phenotype at dpi4 and dpi10. No changes were observed in the anti-inflammatory phenotype at dpi4, but a significant decrease was observed at dpi10 (**Figures 4G, H**). These data indicated that suppression of SFRP2 impedes the functional phenotype transition of macrophages in diabetic wound healing.

We also examined the status of macrophage infiltration and polarization in wound tissues of WT mice. SFRP2 siRNAs increased the macrophage infiltration at both dpi4 and dpi10 and increased inflammatory phenotype at dpi4 (**Supplementary Figures S5A–D**). The ratio of anti-inflammatory phenotype at dpi10 seems to increase, but the statistics analysis showed no difference (**Supplementary Figure S5E**). These data indicated that suppression of SFRP2 may increase the macrophage infiltration through the whole healing process and inflammatory phenotype macrophages at the inflammatory stage of non-diabetic wound healing.

### 3.4 Suppression of SFRP2 affects transcriptome signatures related to energy metabolism in wound tissues

**Figure 5:**
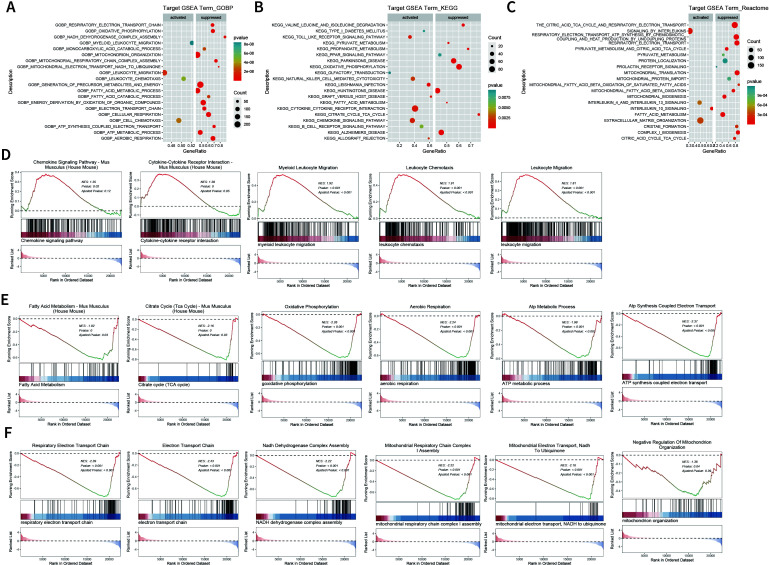
Suppression of SFRP2 affects energy metabolism during wound healing. Wound tissues were collected at dpi4 and subjected to RNA-seq analysis to explore the changes in transcriptome. **(A–C)** GO biological process, KEEG-related pathways, and RACTOME analysis of DEGs based on GSEA analysis. **(D)** Suppression of SFRP2 activates inflammatory response and immune cell chemotaxis and migration. **(E)** Suppression of SFRP2 inhibits energy metabolism (fatty acids metabolism, citrate acid TCA cycle and oxidative phosphorylation, aerobic respiration, ATP metabolic process, and ATP coupled electron transport). **(F)** Suppression of SFRP2 inhibits mitochondrial energy metabolism related to respiratory electron transport chain (respiratory electron transport chain, electron transport chain, NADH dehydrogenase complex assembly, mitochondrial respiratory chain complex I assembly, mitochondrial electron transport, NADH to ubiquinone, and negative regulation of mitochondrion organization).

We further explored the transcriptome changes in wound tissues at dpi4. RNA-seq data showed that a total of 768 differential expressed genes (DEGs) were identified in the SFRP2 siRNAs group, with 263 upregulated genes and 505 downregulated genes (**Supplementary Figures S6A, B**). GO biological process, KEEG-related pathways, and RACTOME of DEGs were analyzed based on GSEA analysis (**Figures 5A–C**). Based on the GSEA analysis, we found that SFRP2 modulates chemokine signaling pathway, cytokine–cytokine receptor interaction, leukocyte chemotaxis and migration, and myeloid leukocyte migration, suggesting that it plays a role in inflammatory response and immune cell chemotaxis and migration (**Figure 5D**). SFRP2 also modulates biological process related to energy metabolism, including fatty acids metabolism, citrate acid TCA cycle and oxidative phosphorylation, aerobic respiration, ATP metabolic process, and ATP coupled electron transport (**Figure 5E**). PPI network analysis also revealed that the core DEGs were energy-metabolism-related genes (**Supplementary Figure S6C**). Further exploration indicated that SFRP2 modulates mitochondrial energy metabolism related to the respiratory electron transport chain. SFRP2 siRNAs suppressed gene sets related to respiratory electron transport chain, electron transport chain, NADH dehydrogenase complex assembly, mitochondrial respiratory chain complex I assembly, mitochondrial electron transport, NADH to ubiquinone, and negative regulation of mitochondrion organization (**Figure 5F**). These data indicate that SFRP2 may be involved in mitochondrial energy metabolism at the early stage of wound healing.

### 3.5 Suppression of SFRP2 compromised the balance between glycolysis and mitochondrial energy metabolism in injury-related macrophages

**Figure 6:**
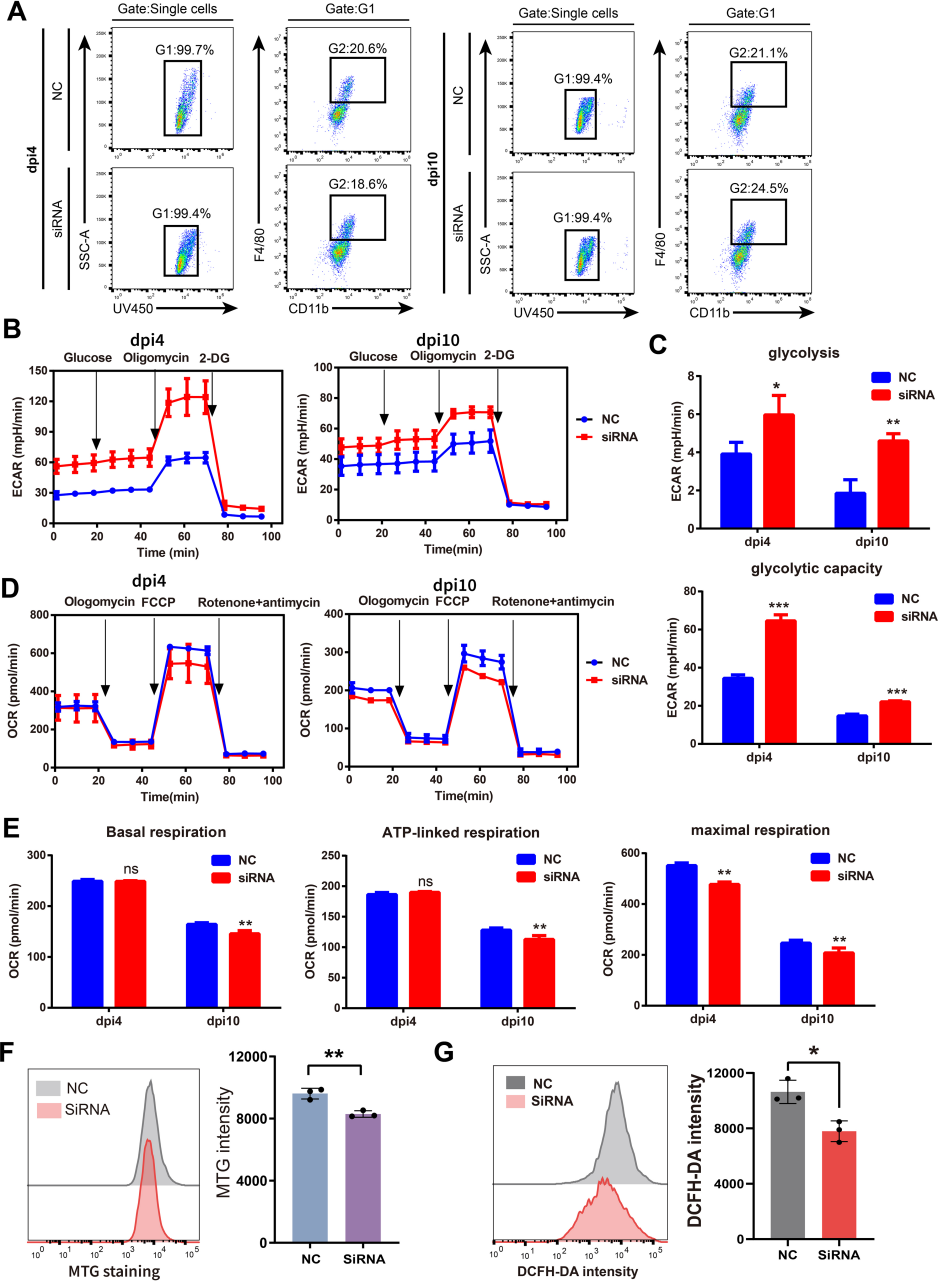
Suppression of SFRP2 compromised the balance between glycolysis and mitochondrial energy metabolism in injury-related macrophages. The injury-associated macrophages were isolated from wound tissues of dpi4 and dpi10. Changes in the oxygen consumption rate (OCR) and the basal extracellular acidification rate (ECAR) were determined. **(A)** Gating strategy of injury-related macrophages. **(B)** ECAR analysis of macrophages. **(C)** The statistics of glycolysis and glycolytic capacity based on ECAR analysis. **(D)** OCR analysis of macrophages. **(E)** The statistics of basal respiration, ATP-link respiration, and maximal respiration based on OCR. **(F)** Flow cytometric analysis of MTG staining. **(G)** Flow cytometric analysis of DCFH staining. Data presented as means ± SD. *p<0.05; **p<0.01; ***p<0.001. ns, non-significant.

We further explored the effects of SFRP2 siRNAs on energy metabolism of injury-related macrophages. The injury-associated macrophages were isolated from wound tissues at dpi4 and dpi10 and then subjected to the extracellular flux analysis (EFA). The gating strategy of cell sorting is shown in **Figure 6A** and **Supplementary Figure S7**. Changes in the oxygen consumption rate (OCR) and the basal extracellular acidification rate (ECAR, a measure of lactate production that reflects glycolysis rate) were determined. SFRP2 siRNAs increased glycolysis and glycolytic capacity of macrophages at both dpi4 and dpi10 (**Figures 6B, C**). A significant reduction in OCR was observed in the SFRP2 siRNAs group at both dpi4 and dpi10. SFRP2 siRNAs reduced basal respiration, ATP production, and respiratory capacity of macrophages (**Figures 6D, E**), suggesting that SFRP2 siRNAs inhibit mitochondrial energy metabolism. A reduction in mitochondrial mass was observed in the SFRP2 siRNAs group as shown by MitoTrackerTM Green (MTG) fluorescent staining and flow cytometric analysis (**Figure 6F**). Moreover, SFRP2 siRNAs decreased ROS level in macrophages (**Figure 6G**). These data indicate suppression of SFRP2 siRNAs decreased mitochondrial energy metabolism and increased glycolysis.

### 3.6 Suppression of SFRP2 affected transcriptome signature related to energy metabolism and inflammatory response and inhibited wnt signaling in inflammatory macrophages

**Figure 7:**
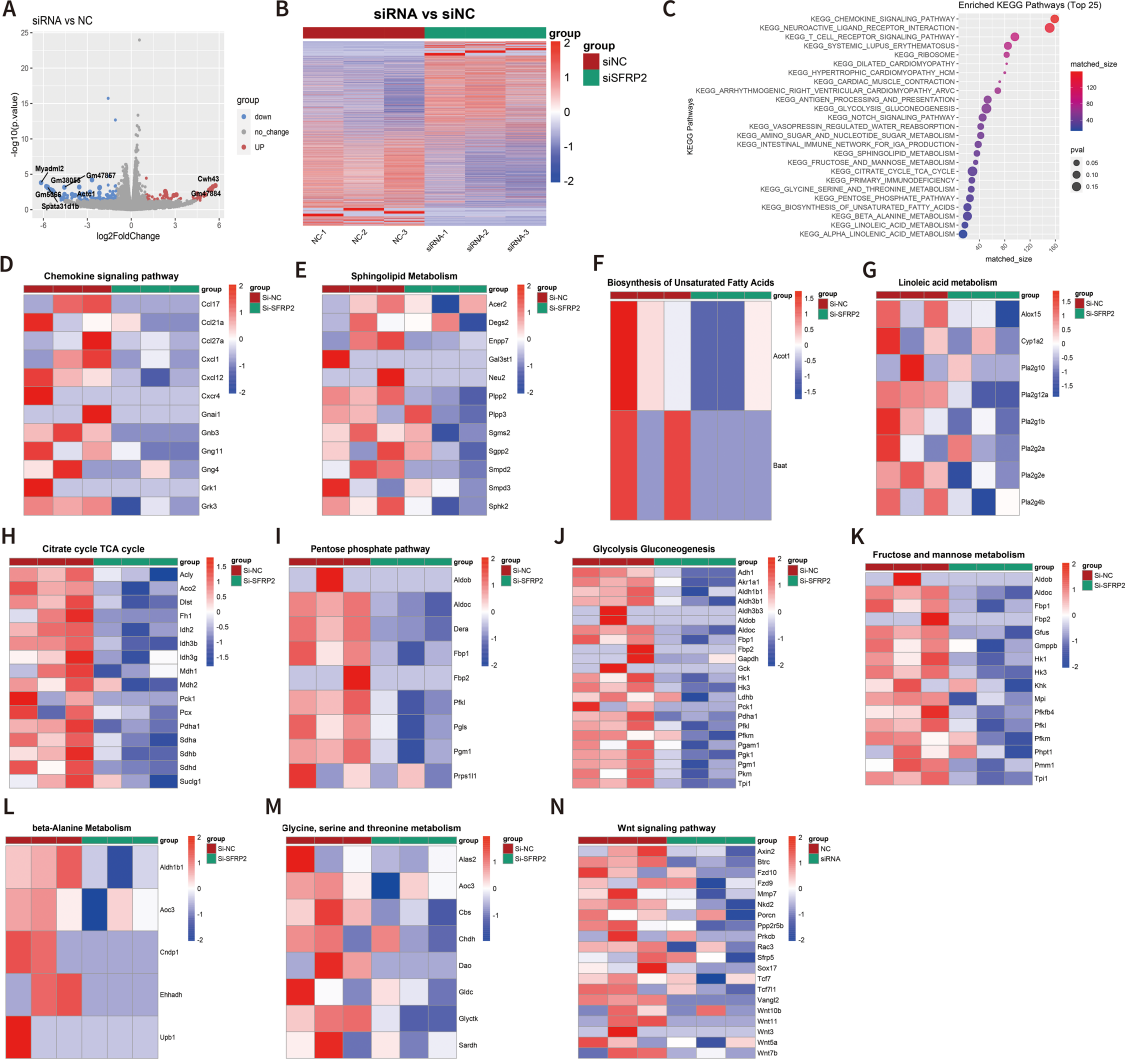
Suppression of SFRP2 affected transcriptome signature related to energy metabolism and inflammatory response and inhibited wnt signaling in inflammatory macrophages. RAW264.7 cells were transfected with siSFRP2 or control siRNAs (NC), polarized by LPS and IFNγ, and subjected to RNA-seq analysis. **(A)** Volcano plot of DEGs. **(B)** Heat map of DEGs. **(C)** KEEG enrichment analysis of DEGs. **(D)** Heat map of chemokine signaling pathway-related DEGs. **(E)** Heat map of sphingolipid-metabolism-related DEGs. **(F)** Heat map of biosynthesis unsaturated fatty-acid-related DEGs. **(G)** Heat map of linoleic acid metabolism-related DEGs. **(H)** Heat map of TCA cycle-related DEGs. **(I)** Heat map of pentose phosphate-related DEGs. **(J)** Heat map of glycolysis and gluconeogenesis-related DEGs. **(K)** Heat map of fructose and mannose metabolism-related DEGs. **(L)** Heat map of beta-alanine metabolism-related DEGs. **(M)** Heat map of glycine, serine, and threonine metabolism-related DEGs. **(N)** Heat map of wnt signaling pathway-related DEGs.

To further explore the molecular mechanism of SFRP2-mediated macrophage polarization and energy metabolism, we analyzed the transcriptome changes in macrophages. RAW264.7 cells were transfected with SFRP2 siRNAs and polarized to inflammatory phenotype using LPS and IFN-γ stimulation. The inflammatory phenotype of RAW 246.7 cells was characterized by increased expression of iNOS and increased accumulation of IL-6 and TNF-α (**Supplementary Figure S8**). RNA-seq analysis was performed to examine the transcriptome of RAW246.7 cells. Data showed that 214 differential expressed genes (DEGs) were identified in the SFRP2 siRNAs group, with 74 upregulated genes and 140 downregulated genes (**Figures 7A, B**). KEGG enrichment analysis showed that DEGs were mainly distributed in metabolism pathway and inflammatory response (**Figure 7C**). The inflammatory-related pathways regulated by SFRP2 siRNAs include chemokine signaling pathways, T-cell receptor signaling, and antigen processing and presentation (**Figure 7D**). The energy metabolism pathways affected by SFRP2 siRNAs include carbohydrate metabolism (citrate cycle TCA cycle, pentose phosphate pathway, glycolysis and gluconeogenesis, fructose, and mannose metabolism), lipids metabolism (sphingolipid metabolism, biosynthesis of unsaturated fatty acids, and linoleic acid metabolism), and amino acid metabolism (glycine, serine and threonine metabolism, and beta alanine metabolism), which consists the three main components of energy metabolism of macrophages (**Figures 7E–M**). In addition, we also analyzed the effect of SFRP2 on wnt signaling. As shown in **Figure 7N**, most of wnt-signaling-related genes were suppressed by SFRP2 siRNA.

### 3.7 Suppression of SFRP2 impeded glycolysis and mitochondrial energy metabolism in inflammatory macrophages

**Figure 8:**
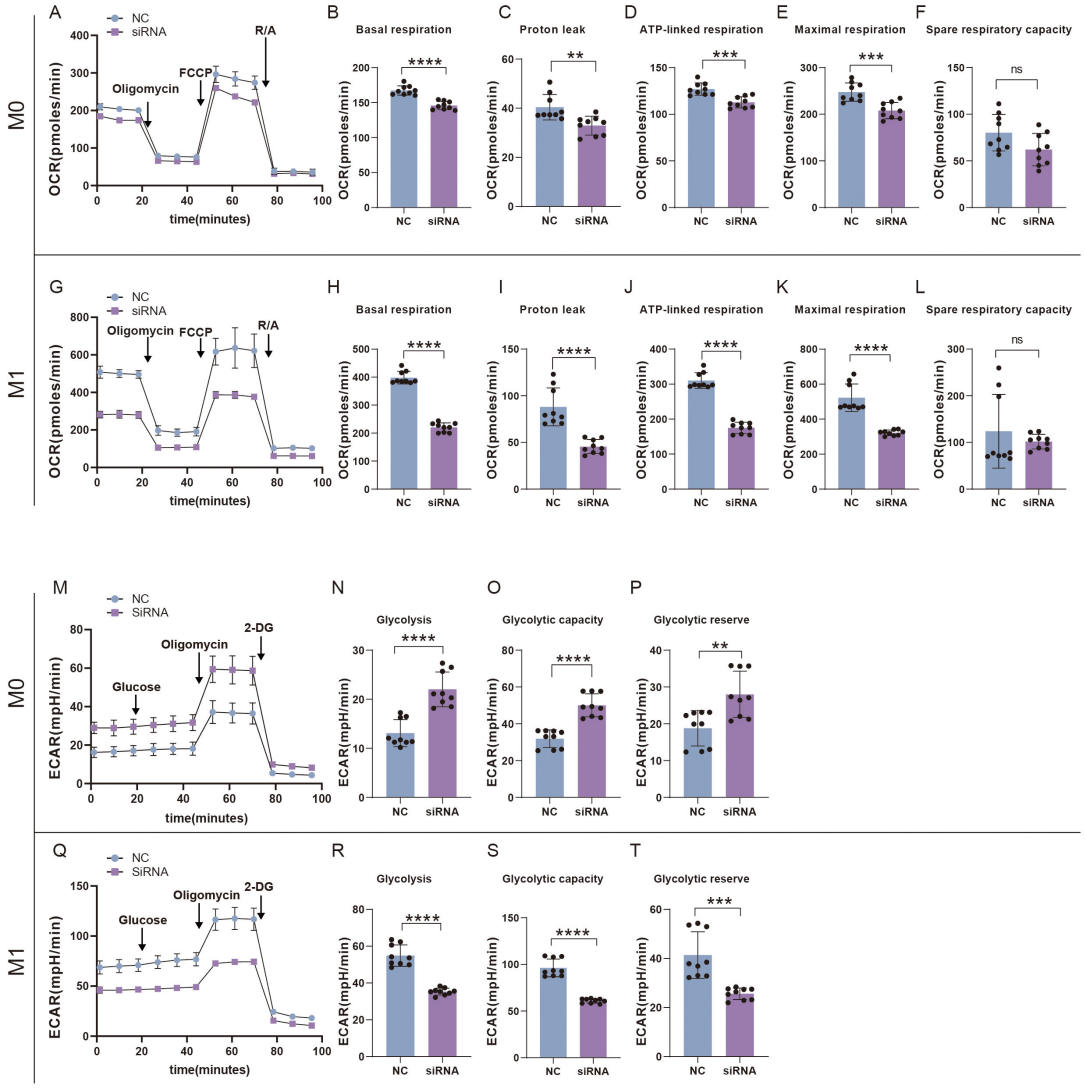
Suppression of SFRP2 impeded glycolysis and mitochondrial energy metabolism in inflammatory macrophages. RAW264.7 cells were transfected with SFRP2 siRNAs and polarized to inflammatory phenotype using LPS and IFN-γ stimulation. The un-polarized phenotype macrophages served as control. ECAR and OCAR were evaluated using EFA analysis. **(A)** OCR analysis of un-polarized macrophages. **(B–F)** The statistics of basal respiration, proton leak, ATP-link respiration, maximal respiration, and spare respiratory capacity based on OCR of un-polarized macrophages. **(G)** OCR analysis of inflammatory macrophages. **(H–L)** The statistics of basal respiration, proton leak, ATP-link respiration, maximal respiration, and spare respiratory capacity based on OCR of inflammatory macrophages. **(M)** ECAR analysis of un-polarized macrophages. **(N–P)** The statistics of glycolysis, glycolytic capacity, and reserve based on ECAR analysis of un-polarized macrophages. **(Q)** ECAR analysis of inflammatory macrophages. **(N–P)** The statistics of glycolysis, glycolytic capacity, and reserve based on ECAR analysis of inflammatory macrophages. Data presented as means ± SD. **p<0.01; ***p<0.001; ****p<0.0001. ns, non-significant.

We also examined the effects of SFRP2 siRNA on glycolysis and mitochondrial energy metabolism of inflammatory macrophages. RAW264.7 cells were transfected with SFRP2 siRNAs and polarized to inflammatory phenotype using LPS and IFN-γ stimulation. The un-polarized macrophages served as control. EFA assays showed that SFRP2 siRNAs suppressed mitochondrial energy metabolism of macrophages, which was characterized by decreased basal respiration, proton leak, ATP-linked respiration, and maximum respiration of macrophages (**Figures 8A–L**). In addition, SFRP2 siRNAs enhanced the total glycolysis of un-polarized macrophages but decreased glycolysis of inflammatory macrophages. As shown in **Figures 8M–T**, SFRP2 siRNAs increased glycolysis, glycolytic capacity, and glycolytic reserve of un-polarized macrophages, and decreased those of inflammatory macrophages. These data indicated that suppression of SFRP2 impeded both glycolysis and mitochondrial energy metabolism in inflammatory macrophages. In un-polarized macrophages, suppression of SFRP2 impeded mitochondrial metabolism but enhanced glycolysis.

### 3.8 Over-expression of SFRP2 enhanced wound healing in diabetic mice

**Figure 9:**
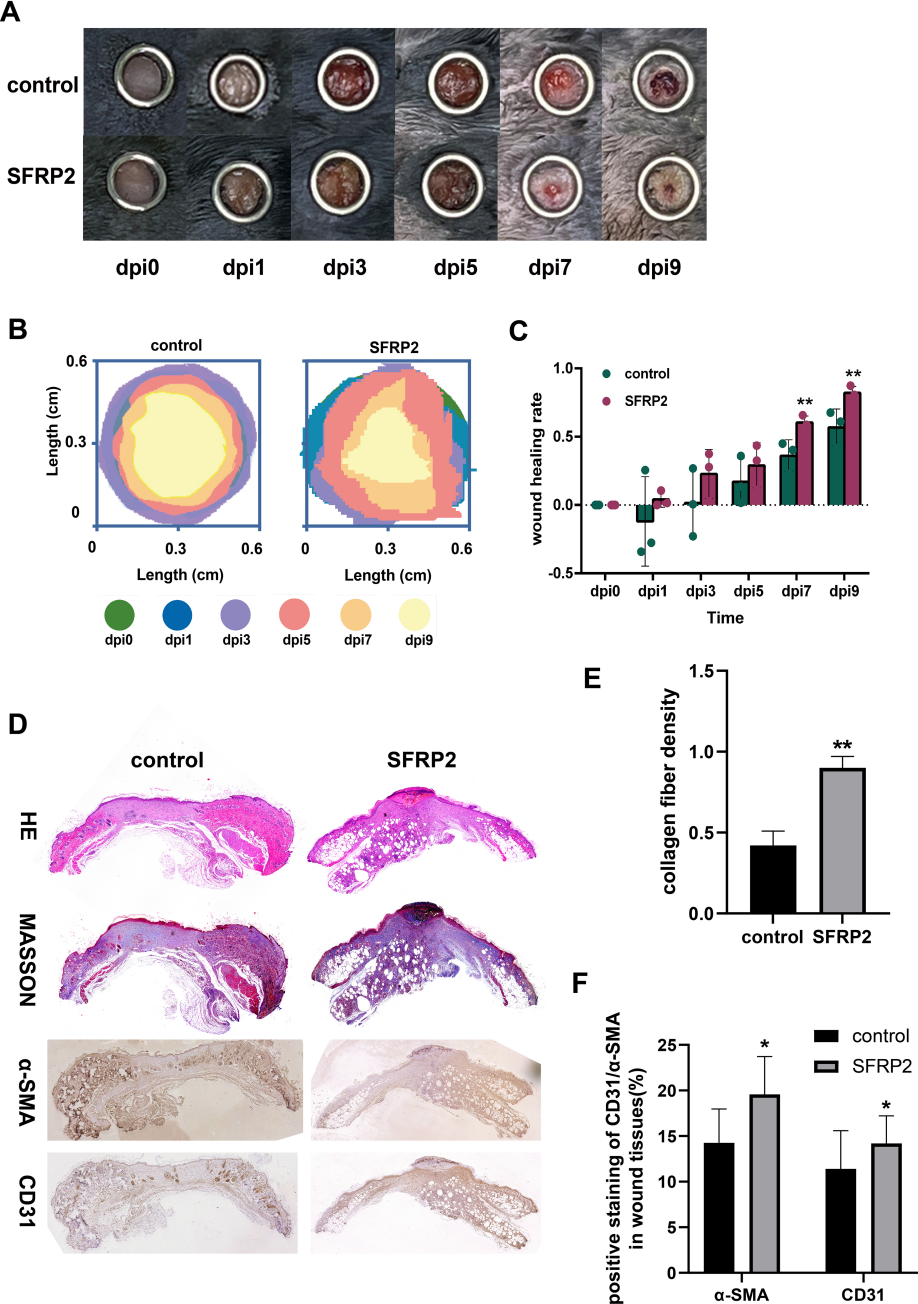
SFRP2 promotes wound healing in diabetic mice. The db/db mice were treated with AAV-SFRP2 or AAV-control. Dorsal full-thickness skin wounds were created by 6-mm punch biopsies after 14 days. The wound healing was evaluated at indicated time points. **(A)** Representative images of wound healing. **(B)** Measurement of wound healing by ImageJ. **(C)** The statistics of wound healing rate. **(D)** Representative images of HE, MASSON staining, and IHC staining of CD31 and α-SMA. **(E)** The statistics of collagen fiber density based on MASSON staining. **(F)** The statistics of angiogenesis and matrix deposition based on IHC staining. Data presented as means ± SD. *p<0.05; **p<0.01.

To evaluate the therapeutic potential of SFRP2 in diabetic wound healing, AAV-SFRP2 or AAV-control was injected at the dorsal skin of db/db mice 14 days before skin wounds were created. As shown in **Figures 9A–C**, AAV-SFRP2 enhanced the wound healing process of diabetic mice. HE and MASSON staining showed higher collagen fiber density in wound tissues of AAV-SFRP2-treated mice (**Figures 9D, E**). IHC staining of α-SMA and CD31 demonstrated better matrix remodeling and angiogenesis in wound tissues of AAV-SFRP2-treated mice (**Figures 9D, F**). Collectively, these data indicated over-expression of SFRP2 enhanced wound healing in diabetic mice.

## 4 Discussion

In this study, we identified five potential target genes, SFRP2, KIT, FGFR2, AREG, and SPRR1B. FGFR2, AREG, and SPRR1B have been implicated in wound healing and tissue regeneration, which also demonstrates the effectiveness of our screening method. FGF, the ligand of FGFR, has been recommended by the American Wound Healing Association and the European Wound Management Association for the treatment of refractory ulcers. AREG, also known as amphiregulin, could be induced by tissue-resident immune cells to promote tissue integrity and wound repair (33). It has been reported that basophil-derived AREG would worsen chronic allergic skin inflammation (34). It also plays an important role on the recruitment and repair function of MAIT cells during skin wound healing (29). SPRR1B, also known as SPRR1 and cornifin, encodes a precursor of the keratinocyte cornified envelope (35). It plays a certain role in the differentiation of normal keratinocytes, which is a critical cell population in skin re-epithelialization during wound healing (36). KIT, also known as C-Kit and CD117, is a proto-oncogene and receptor tyrosine kinase. Upon activation by its cytokine ligand, stem cell factor (SCF), this protein phosphorylates multiple intracellular proteins that play a role in the proliferation, differentiation, migration, and apoptosis of many cell types and thereby plays an important role in the involvement of stem cells and mast cell in wound healing (37–40). We chose SFRP2 as the study subject for several reasons: (i) the role of SFRP2 in refractory wound has never been reported; (ii) SFRP2 was upregulated in oral mucus with strong healing capacity and downregulated in non-healing DFU, which suggested that the expression of SFRP2 may be associated with healing capacity of DFU; and (iii) the expression of SFRP2 may be associated with immune cells infiltration of DFU.

*In vivo* study showed that suppression of SFRP2 impeded angiogenesis and matrix remodeling and delayed the wound healing process of diabetic mice (**Figure 2**), and treatment of AAV-SFRP2 augmented wound healing in diabetic mice and demonstrated the therapeutic potential of SFRP2. Neutrophils and macrophages are the major immune cell populations involved in wound healing. Suppression of SFRP2 did not affect neutrophils infiltration but increased macrophage infiltration. It is reasonable to conclude that macrophages are the main target of SFRP2. Further evidence demonstrates that in diabetic mice treated with SFRP2 siRNAs, inflammatory macrophages were maintained at a high level through the whole wound healing process, and anti-inflammatory macrophages were decreased at the later stage of wound healing (**Figure 4**). This finding indicates that suppression of SFRP2 impedes the functional phenotype transition of macrophages at the later stage of wound healing process. The transition of pro-inflammatory macrophages to healing-favored anti-inflammatory macrophages is important for wound healing (5). Inflammatory macrophages are responsible for eliminating pathogens at the early stage of wound healing, and anti-inflammatory macrophages function as pro-healing immune cells at the later stage of wound healing (4, 5). Deficiency of anti-inflammatory macrophages disturb the healing process and impede angiogenesis and matrix remodeling at the later stage of wound healing (4, 5). It is reasonable to conclude that disruption of the inflammatory-to-anti-inflammatory transition may have contribute to the delayed wound healing caused by SFRP2 deficiency. However, SFRP2 may play a different role in non-diabetic wound healing. In control mice, suppression of SFRP2 may increase the macrophage infiltration through the whole healing process and maintain a high level of inflammatory macrophages at the inflammatory stage (**Supplementary Figure S5E**).

One of the critical findings is that SFRP2 is involved in the metabolic reprogramming of macrophages. We first found that suppression of SFRP2 affects transcriptome signature related to the energy metabolism in wound tissue. SFRP2 siRNAs suppressed gene sets related to fatty acids metabolism, citrate acid TCA cycle and oxidative phosphorylation, aerobic respiration, ATP metabolic process, and ATP-coupled electron transport (**Figure 5E**). Further exploration suggests that SFRP2 modulates mitochondrial energy metabolism, which is characterized by respiratory electron transport chain, NADH dehydrogenase complex assembly, mitochondrial respiratory chain complex I assembly, mitochondrial electron transport, NADH to ubiquinone, and negative regulation of mitochondrion organization (**Figure 5F**). Given that SFRP2 plays a role in macrophage infiltration and polarization, we assume that the role of SFRP2 may be related to energy metabolism. Aerobic glycolysis dominates inflammatory phenotype macrophages, while oxidative phosphorylation is favored by anti-inflammatory phenotype (6, 41). EFA analysis of injury-related macrophages demonstrates that suppression of SFRP2 impeded mitochondrial energy metabolism as evidenced by decreased oxygen consumption rate, ROS, and mitochondrial mass at both dpi4 and dpi10 (**Figures 6D–G**). As compensation, glycolysis plays a dominant role in injury-related macrophages, which is characterized by increased glycolysis and glycolytic capacity at both dpi4 and dpi10 (**Figures 6B, C**). In addition, the suppression of SFRP2 affected transcriptome signature related to energy metabolism and inflammatory response in inflammatory macrophages (**Figure 7**), which at least partially supports our hypothesis that SFRP2 modulates the function of macrophages through regulating the balance between mitochondrial metabolism and glycolysis. However, SFRP2-mediated regulation of energy metabolism in immortalized RAW264.7 cells seems a little bit different from those of injury-related macrophages isolated from diabetic mice. The findings in RAW264.7 indicated that suppression of SFRP2 impeded both glycolysis and mitochondrial energy metabolism in inflammatory macrophages. In un-polarized macrophages, the suppression of SFRP2 impeded mitochondrial metabolism but enhanced glycolysis. We hypothesize that the reason for this discrepancy is that the energy metabolism of immortalized cells is different from those of primary cells.

There is also a noteworthy finding about the effect of SFRP2 on wnt signaling pathway. As shown in **Figure 7N**, most of the wnt-signaling-related genes were suppressed by SFRP2 siRNA. The traditional view proposes that SFRP2 is a negative regulator of wnt signaling pathway (21, 42, 43). However, a few studies propose a different theory that SFRP2 may augment wnt signaling pathway (44–46). Our data favor the role of SFRP2 as a positive regulator of wnt signaling pathway. We speculate that SFRP2 may exhibit different functions under different diseases and physiological conditions, and the association between SFRP2 and wnt signaling may change under different conditions. There are a few questions that need to be clarified in order to explain the mechanism. SFRP2 is an extracellular protein. There is a strong possibility that SFRP2 binds to a receptor protein to activate or suppress signaling pathway. It is crucial to clarify which protein is the receptor of SFRP2 and which protein is the downstream effector of SFRP2-mediated signaling transduction. Based on previous reports, there is equal possibility that SFRP2 binds to wnt proteins and Frizzled proteins (Fzds) (47, 48). PLA assays showed that SFRP2 binds to wnt3a and wnt5a (**Supplementary Figure S9**). We did not examine the binding between SFRP2 and Fzds yet. The mammalian Frizzled subfamily has 10 members (Fzd1 to Fzd10), and they may mediate signaling through different pathways. In the canonical wnt/β-catenin pathway, wnt proteins bind to Fzd and the co-receptors LRP5 or LPR6 and activate wnt/β-catenin pathway through inhibiting phosphorylation of β-catenin by GSK3-β. Fzds can also bind to other secreted proteins, such as Norrin and R-Spondin (49–51). There is great possibility that SFRP2 binds to Fzds and activate wnt signaling pathway. Unfortunately, we cannot provide relevant evidence yet. However, we will continue to dig deeper into the relevant mechanism in the future study.

In summary, our findings help decipher the role of SFRP2 in the transition of macrophage functional phenotypes and in diabetic wound healing. The theory proposed in this work provides solid support for developing therapy for diabetic wounds.

## Data Availability

The datasets presented in this study can be found in online repositories. The names of the repository/repositories and accession number(s) can be found below: GSE268759 and GSE268760 (GEO).
