# Supplementary material for: SFRP2 modulates functional phenotype transition and energy metabolism of macrophages during diabetic wound healing
Source: Front Immunol. 2024 Oct 11;15:1432402. doi: 10.3389/fimmu.2024.1432402 (PMC11502328; doi:10.3389/fimmu.2024.1432402)
Supplement: Supplementary file 1 [file DataSheet1.docx]

Supplementary Material

# Supplementary Figures


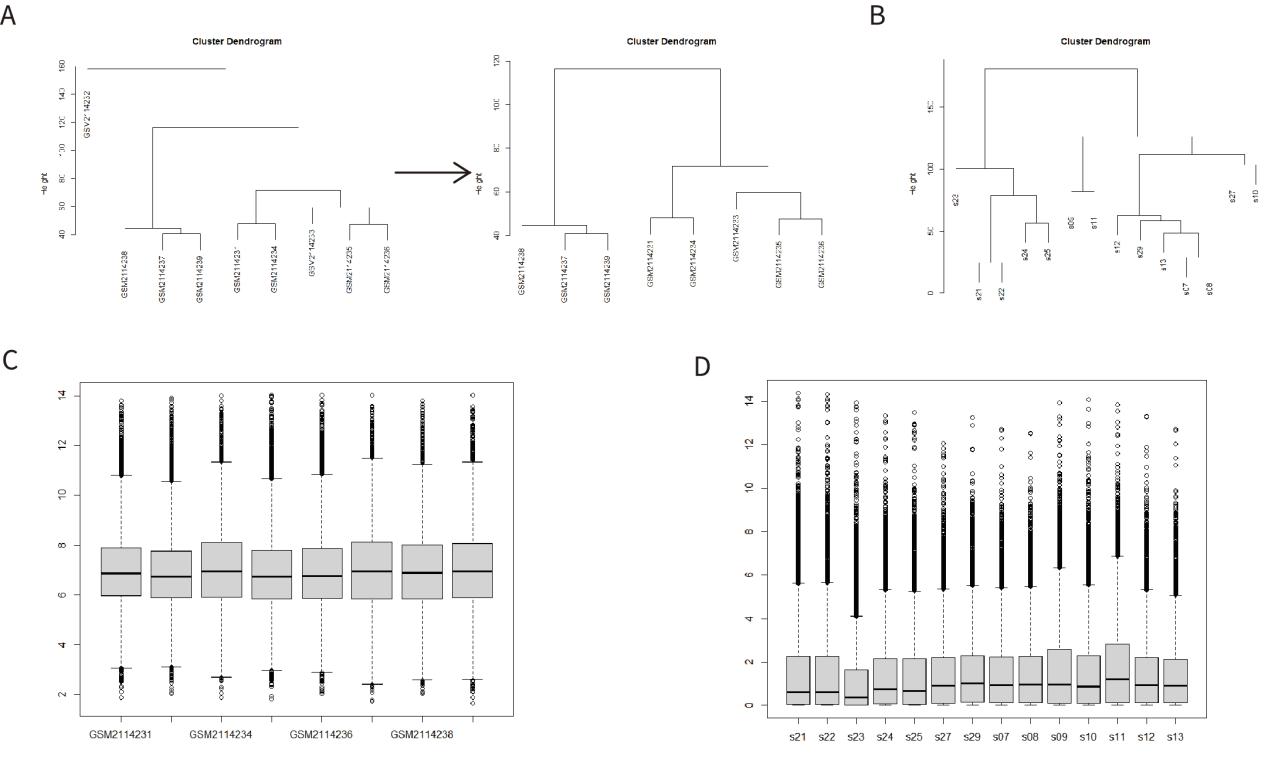


**Supplementary figure 1** Data processing of GSE80178 and GSE134431. A, Cluster tree diagram of GSE80178. B, Cluster tree diagram of GSE134431. C, box plots of GSE80178. D, box plots of GSE134431.


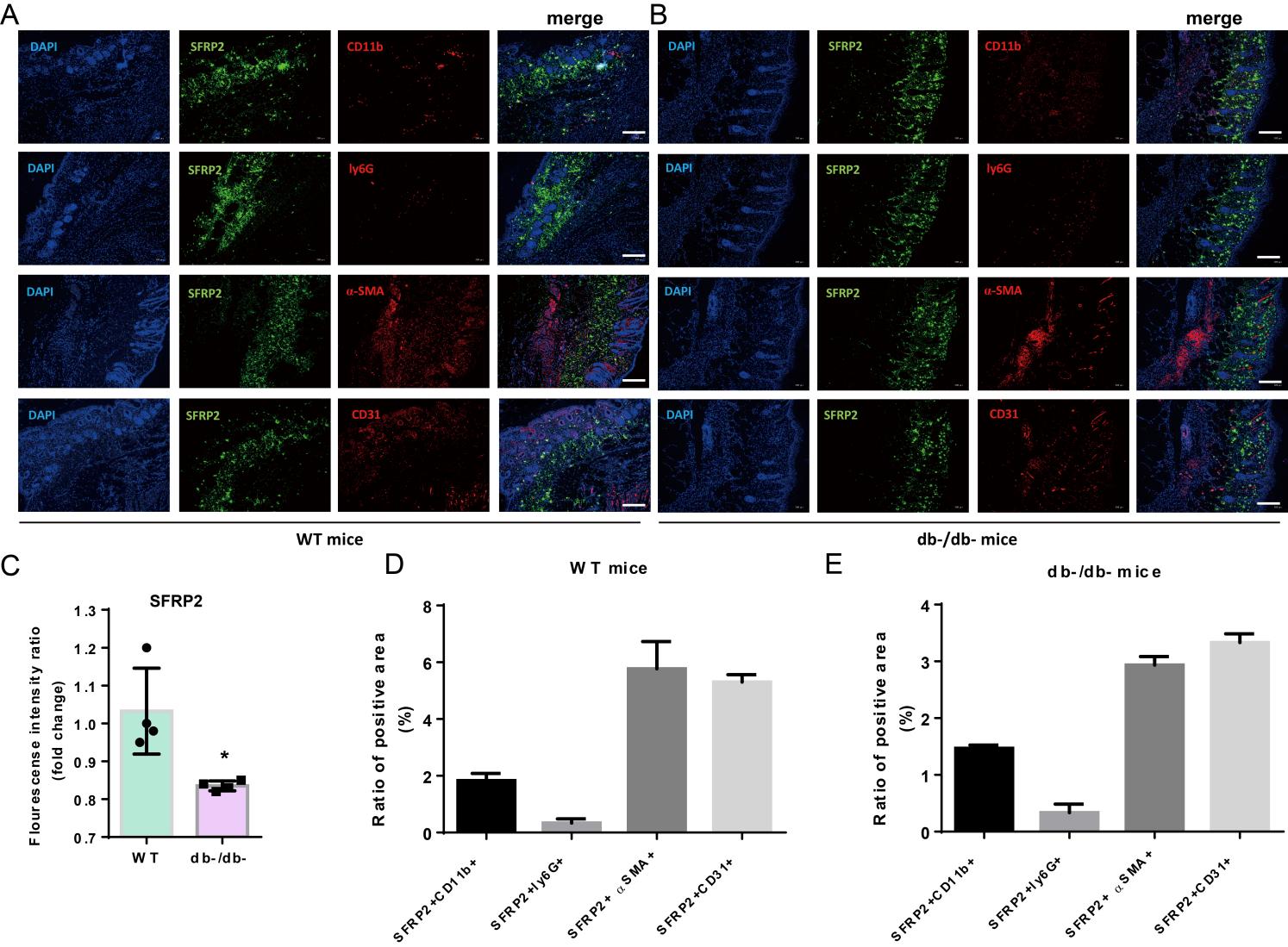


**Supplementary figure 2**  The expression profile of SFRP2 in the main cell populations of skin tissue. The expression profile of SFRP2 in the endothelial cells, fibroblasts, neuthrophils and macrophages of skin tissues were determined by IF staining. The endothelial cells, fibroblasts, neuthrophils and macrophages in skin tissues of WT mice (A) and db/db mice (B) were marked by their corresponding marker CD31, α-SMA, ly6G and CD11b (indicated by red staining). The SFRP2 proteins was marked by green staining. C, the accumulation of SFRP2 proteins in WT and db/db mice were calculated based on flourescense intensity. D-E, the accumulation of SFRP2 proteins in the main cell populations (endothelial cells, fibroblasts, neuthrophils and macrophages) of WT and db/db mice.


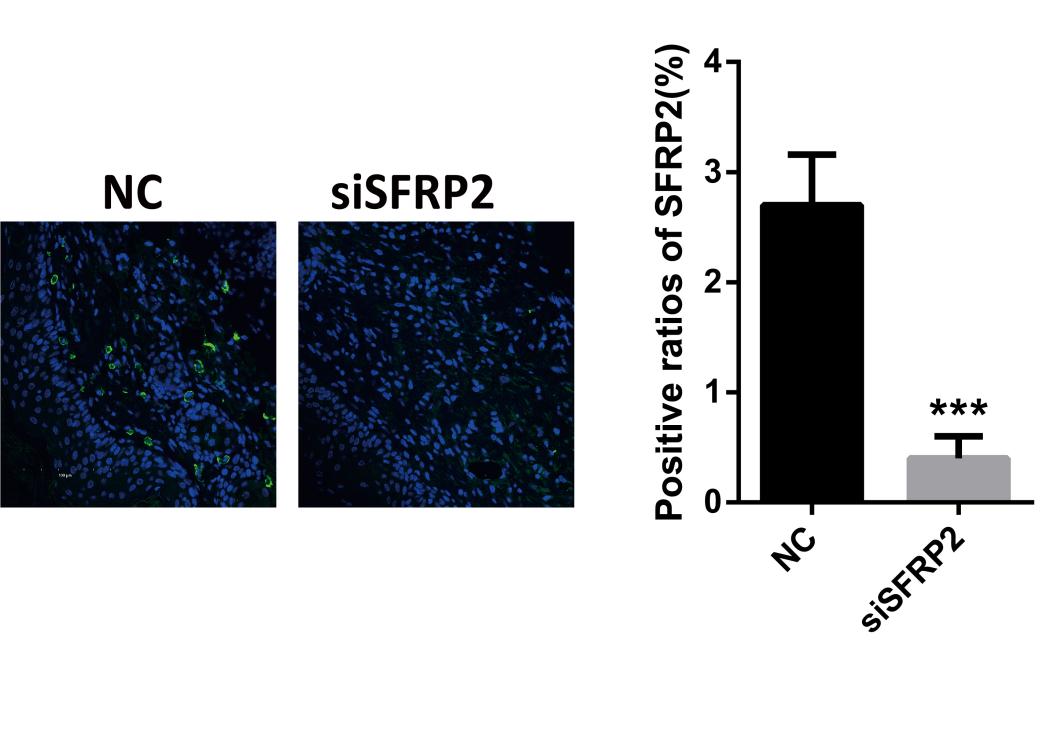


**Supplementary figure 3** IF staining of SFRP2 in wound tissues of diabetic mice treated with SFRP2 siRNAs and control siRNAs(NC) at dpi3.


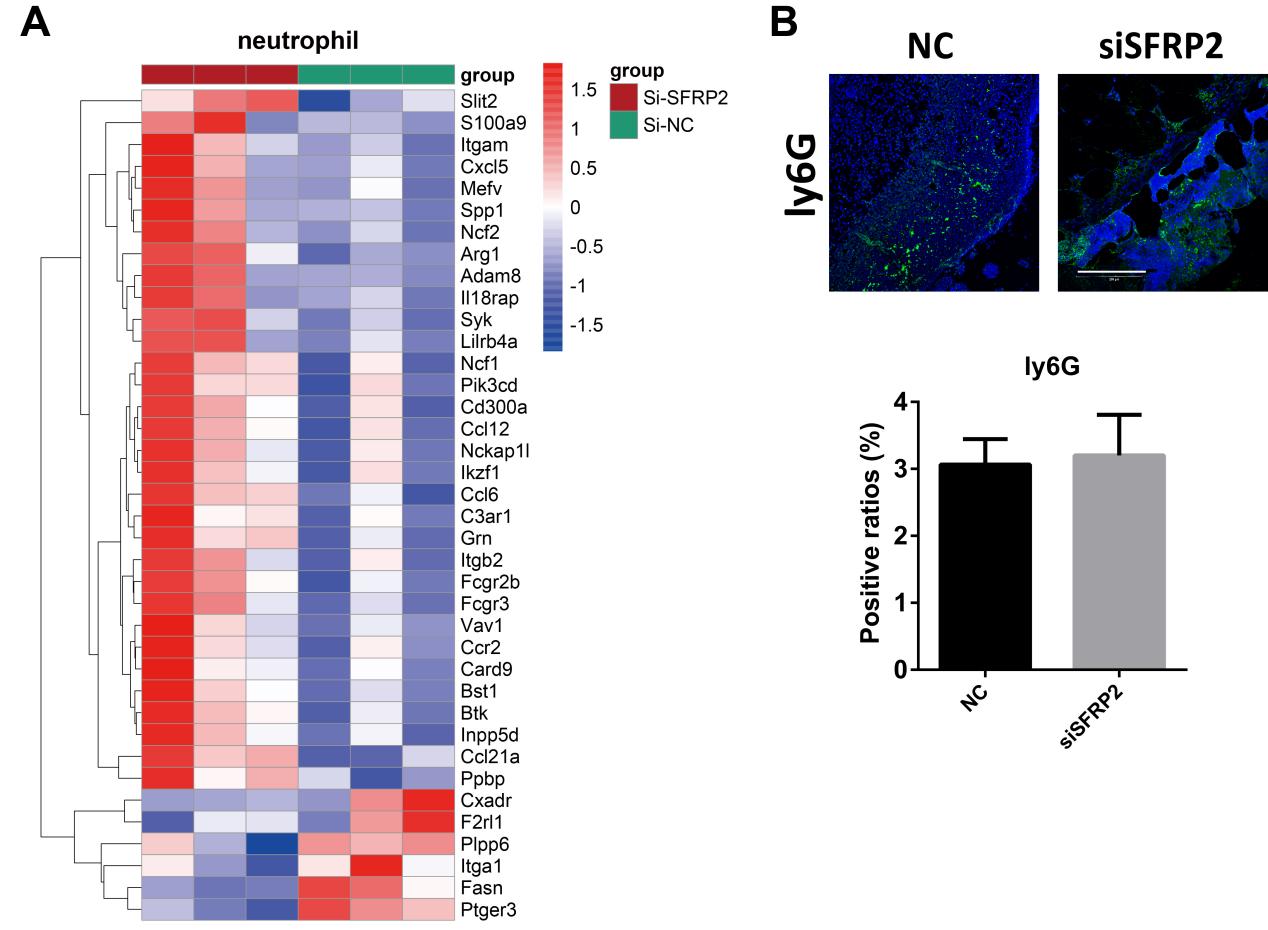


**Supplementary figure 4** The effects of SFRP2 siRNA on neutrophils infiltration. A, heat map of neutrophils-related genes based on RNA-seq analysis of wound tissues at dpi4. B, IF staining of neutrophils marker ly6G at dpi4.


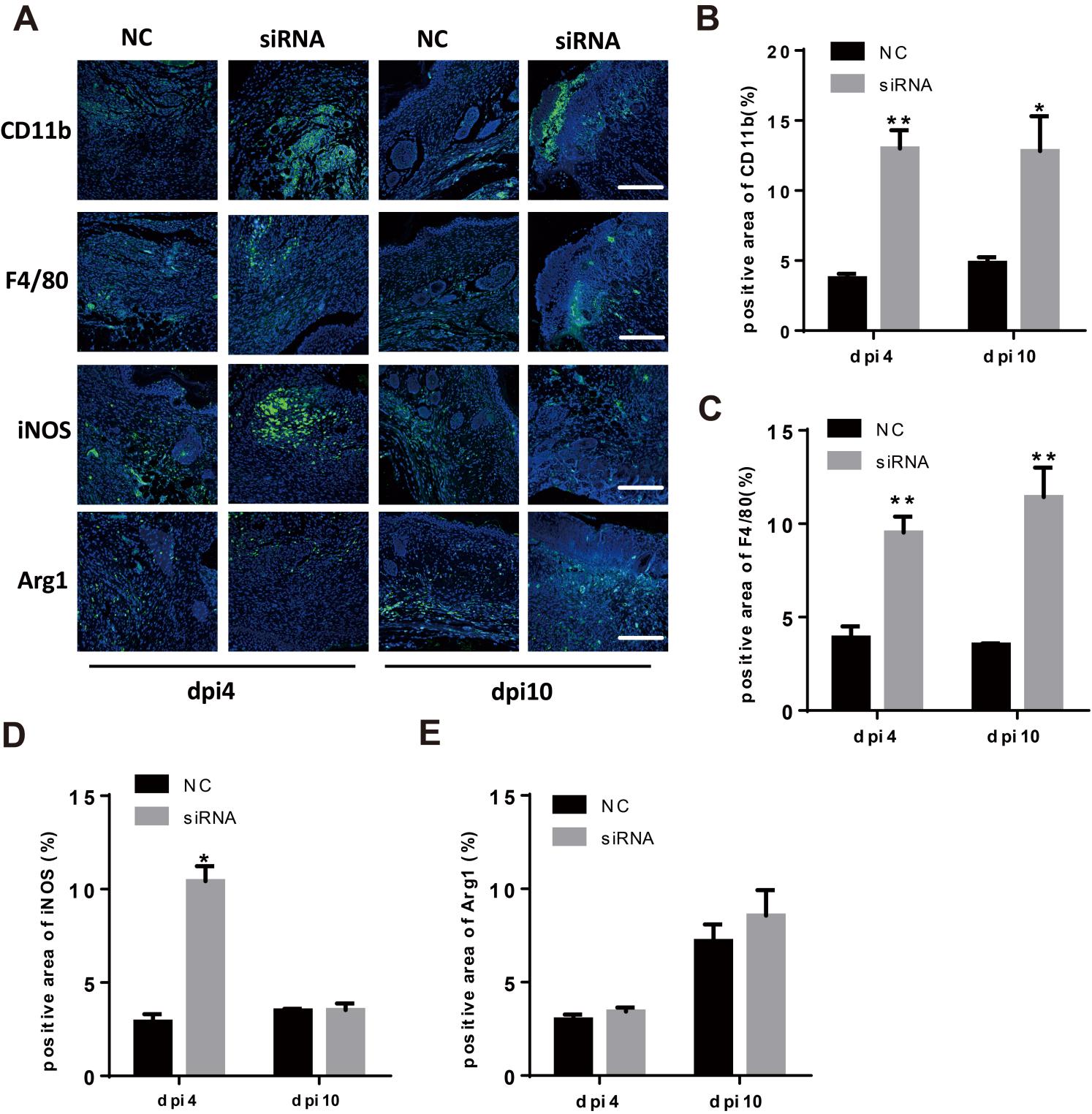


**Supplementary figure 5** The status of macrophage infiltration and polarization in wound tissues of WT mice. Macrophage infiltration was characterized by CD11b and F4/80 staining. The inflammatory and anti-inflammatory phenotype were indicated by iNOS and Arg1, respectively. A, IF staining of CD11b, F4/80, iNOS and Arg1. Bar=200μm. B-E, the statistics of positive staining marked by CD11b, F4/80, iNOS and Arg1. Data presented as means±sd. *, P<0.05; **, P<0.01.


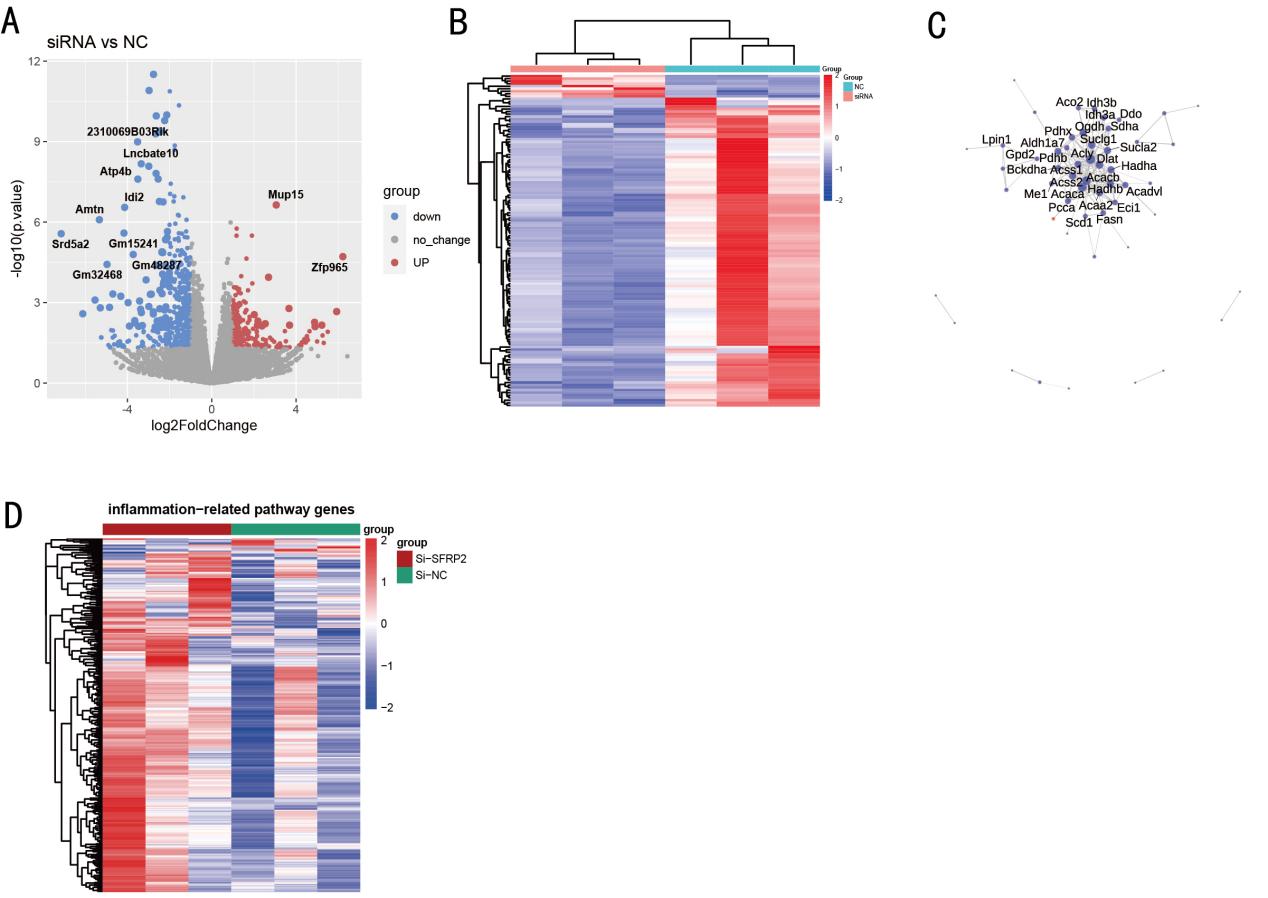


**Supplementary figure 6** RNA-seq analysis of wound tissues at dpi4. Wound tissues were collected at dpi4 and subjected to RNA-seq analysis to explore the changes of transcriptome. The status of immune cell infiltration was analyzed using CIBERSORT algorithm. A, volcano plot of DEGs. B, heat map of DEGs. C, PPI network of DEGs. D, heat map of inflammation-related DEGs.


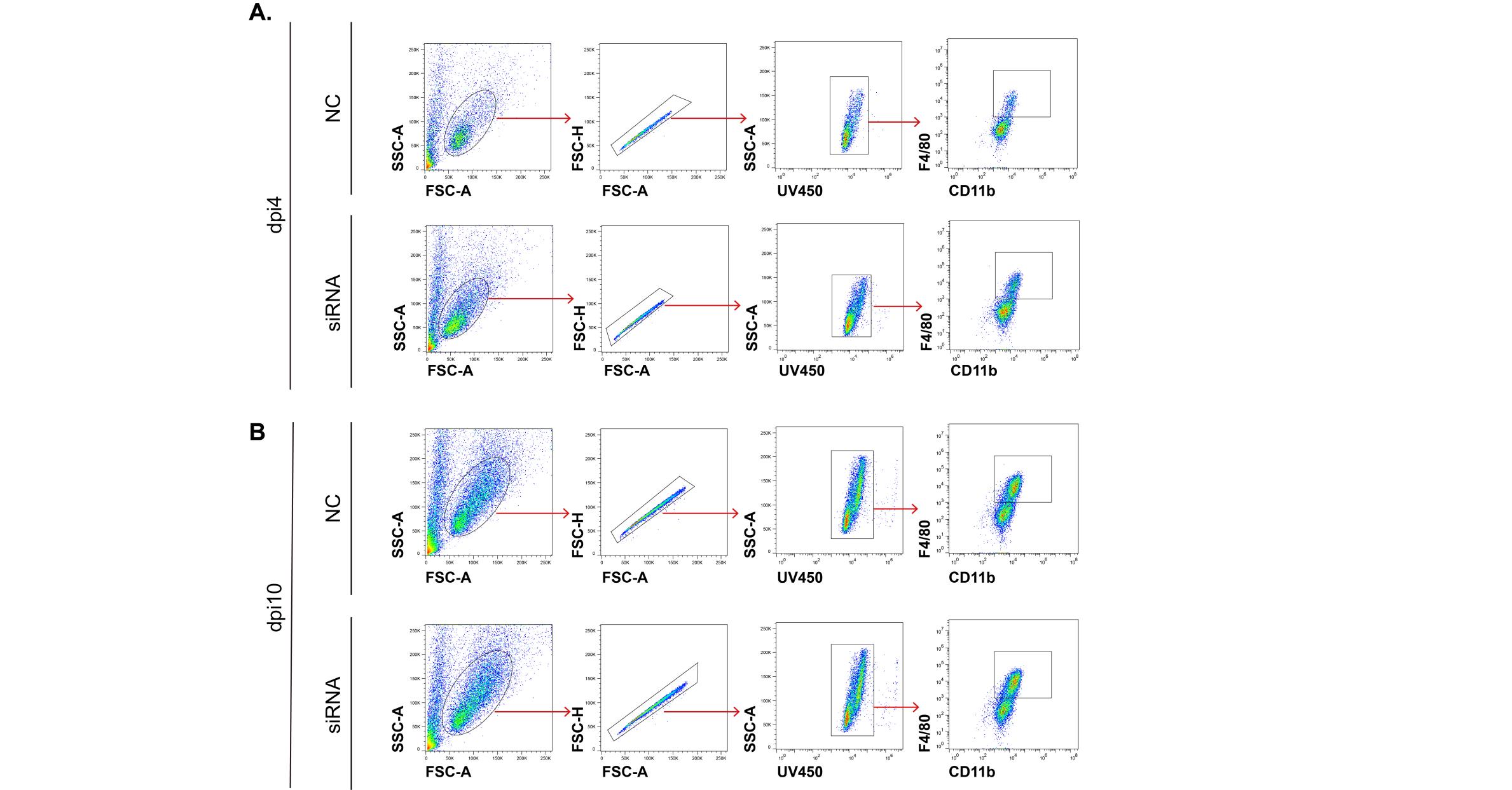


**Supplementary figure 7 Gating strategy of injury-related macrophages.** The injury-associated macrophages were isolated from wound tissues of dpi4 (A) and dpi10 (B). Cells were stained with a Fixable LIVE/DEAD viability dye to exclude dead cells. Monoclonal antibodies for surface staining included: APC-Cyanine7-conjugated anti-CD11b, PE--Cyanine5-conjugated anti-F4/80.


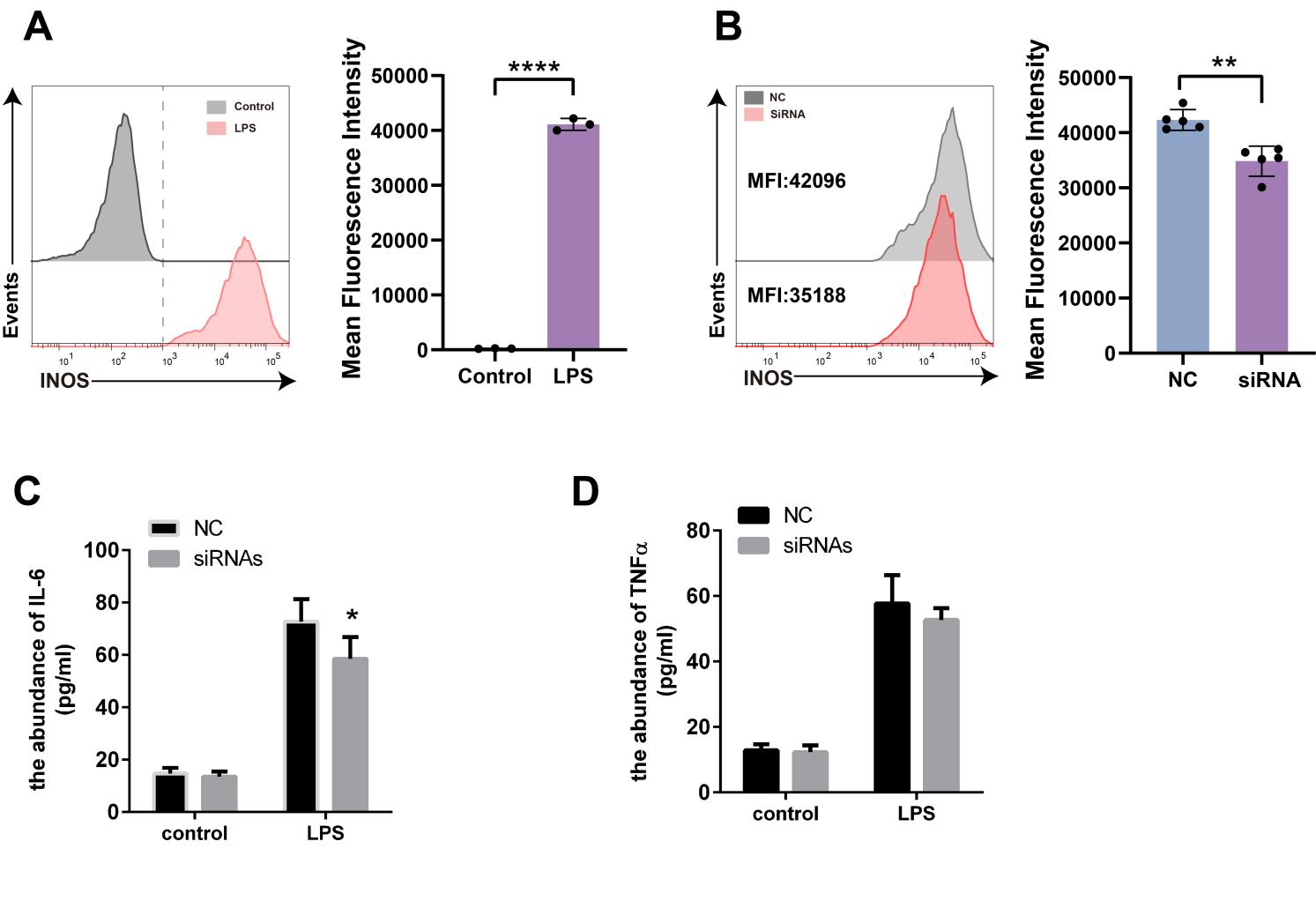


**Supplementary figure 8** Characterization of inflammatory phenotype of RAW264.7 cells after LPS stimulation. RAW264.7 cells were treated with LPS (100ng/ml) and IFN-γ (20ng/ml) for 24h. The inflammatory phenotype of RAW 246.7 cells was characterized by increased expression of iNOS and increased accumulation of IL-6 and TNF-α. A, flow cytometry analysis of iNOS in cells treated with or without LPS and IFN-γ. B, flow cytometry analysis of iNOS in cells transfected with NC or siRNAs. C-D, the abundance of IL6 and in culture mediums of RAW264.7 cells were determined by ELISA.


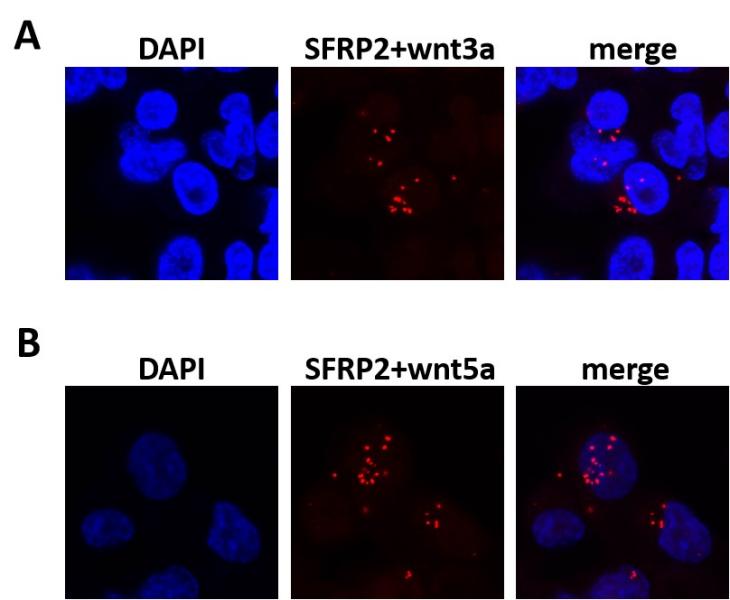


**Supplementary figure 9** SFRP2 bind to wnt3a and wnt5a in RAW264.7 cells. PLA assays was performed to examine the binding between SFRP2 and wnt3a or wnt5a. The red spots indicated positive binding between SFRP2 and wnt3a or wnt5a. DAPI was used to stain the nuclei.

# Supplementary Tables

Table 1 antibodies for IHC staining, IF staining and flow cytometry

| NO. | antibodies | Brand and catalog number | Dilution |
| --- | --- | --- | --- |
| 1 | Anti-SFRP2-antibody (rabbit) | Proteintech, 66328-1-PBS | IHC, 1:500; IF, 1:200  PLA, 1:200 |
| 2 | Anti-SFRP2-antibody (mouse) | Proteintech, 66328-1-Ig | IHC, 1:200  IF: 1:200 |
| 3 | Anti-CD31-antibody (rabbit) | Proteintech, 11265-1-AP | IHC, 1:200  IF: 1:100 |
| 4 | Anti-α-SMA-antibody (rabbit) | Proteintech, 14395-1-AP | IHC, 1:100  IF: 1:50 |
| 5 | Anti-CD11b-antibody (rabbit) | Abcam, ab133357 | IF, 1:100 |
| 6 | Anti-F4/80-antibody (mouse) | SantaCruz, sc-377009 | IF, 1:50 |
| 7 | Anti-CD207-antibody (mouse) | Proteintech, 67788-1-Ig | IF, 1:4000 |
| 8 | Anti-iNOS-antibody (rabbit) | Proteintech, 80517-1-RR | IF, 1:500 |
| 9 | Anti-Arg1-antibody (rabbit) | Proteintech, 16001-1-AP | IF, 1:500 |
| 10 | DyLight 488, Goat Anti-Mouse IgG | Abbkine, A23210 | IF, 1:200 |
| 11 | Dylight 488, Goat Anti-Rabbit IgG | Abbkine, A23220 | IF, 1:200 |
| 12 | Rat monoclonal anti-CD11b-antibody | eBioscience, 11-0112-82 | Flow: 1:300 |
| 13 | Rat monoclonal anti-F4/80 | eBioscience, 17-4801-82 | Flow: 1:300 |
| 14 | LIVE/DEAD™ Fixable Blue Dead Cell Stain Kit | Invitrogen, L34961 | Flow: 1:1000 |
| 15 | Wnt3 antibody mouse | Santa cruz sc74537 | PLA: 1:50 |
| 16 | Wnt5 antibody mouse | Santa cruz sc23698 | PLA: 1:50 |

Table 2 siRNAs used in this study

| ID | sequence |  |
| --- | --- | --- |
| SiRNA-3 | UCAACAGAGACACCAAGAUdTdT | Sense |
|  | AUCUUGGUGUCUCUGUUGAdTdT | Anti-sense |
| SiRNA-2 | CCAAGAAUGAGGACGACAAdTdT | Sense |
|  | UUGUCGUCCUCAUUCUUGGdTdT | Anti-sense |
| SiRNA-1 | GGAUUCCGCUGGUCAUGAAdTdT | Sense |
|  | UUCAUGACCAGCGGAAUCCdTdT | Anti-sense |
| NC | UUCUCCGAACGUGUCACGUdTdT | Sense |
|  | ACGUGACACGUUCGGAGAAdTdT | Anti-sense |
